# Genome-Wide Identification and Characterization of *R2R3MYB* Family in *Cucumis sativus*

**DOI:** 10.1371/journal.pone.0047576

**Published:** 2012-10-23

**Authors:** Qiang Li, Cunjia Zhang, Jing Li, Lina Wang, Zhonghai Ren

**Affiliations:** State Key Laboratory of Crop Biology, Key Laboratory of Biology and Genetic Improvement of Horticultural Crops (Huanghuai Region), Ministry of Agriculture, College of Horticulture Science and Engineering, Shandong Agricultural University, Tai’an, People’s Republic of China; Michigan State University, United States of America

## Abstract

**Background:**

The R2R3MYB proteins comprise one of the largest families of transcription factors in plants. Although genome-wide analysis of this family has been carried out in some species, little is known about *R2R3MYB* genes in cucumber (*Cucumis sativus* L.).

**Principal Findings:**

This study has identified 55 *R2R3MYB* genes in the latest cucumber genome and the *CsR2R3MYB* family contained the smallest number of identified genes compared to other species that have been studied due to the absence of recent gene duplication events. These results were also supported by genome distribution and gene duplication analysis. Phylogenetic analysis showed that they could be classified into 11 subgroups. The evolutionary relationships and the intron - exon organizations that showed similarities with *Arabidopsis*, *Vitis* and *Glycine* R2R3MYB proteins were also analyzed and suggested strong gene conservation but also the expansions of particular functional genes during the evolution of the plant species. In addition, we found that 8 out of 55 (∼14.54%) cucumber *R2R3MYB* genes underwent alternative splicing events, producing a variety of transcripts from a single gene, which illustrated the extremely high complexity of transcriptome regulation. Tissue-specific expression profiles showed that 50 cucumber *R2R3MYB* genes were expressed in at least one of the tissues and the other 5 genes showed very low expression in all tissues tested, which suggested that cucumber *R2R3MYB* genes took part in many cellular processes. The transcript abundance level analysis during abiotic conditions (NaCl, ABA and low temperature treatments) identified a group of *R2R3MYB* genes that responded to one or more treatments.

**Conclusions:**

This study has produced a comparative genomics analysis of the cucumber *R2R3MYB* gene family and has provided the first steps towards the selection of *CsR2R3MYB* genes for cloning and functional dissection that can be used in further studies to uncover their roles in cucumber growth and development.

## Introduction

The MYB family of proteins is large, functionally diverse and represented in all eukaryotes. Most MYB proteins function as transcription factors with MYB binding domain conferring the ability to bind DNA [1]. The *MYB* gene family is divided into different types according to the number of repeat(s) in the MYB domain: *4RMYB* has four repeats, *3RMYB* (*R1R2R3MYB*) has three consecutive repeats, *R2R3MYB* has two repeats, and the MYB-related type usually, but not always, has a single repeat [1], [2], [3], [4]. However, most plant *MYB* genes encode R2R3MYB class proteins, which containing two repeats [1], [5]. Each of these MYB repeats contains three-helices, with the second and third helices forming a helix-turn-helix structure when bound to DNA [6]. Moreover, R2R3MYB proteins are characterized by the presence of a conserved MYB domain and a highly variable C-terminal region. The C-terminal region is responsible for establishing protein-protein interactions with other components [7], [8].

Based on their well conserved DNA-binding domains, *R2R3MYB* family have been annotated genome-wide in *Arabidopsis* (126 members) [5], *Oryza sativa* (102 members) [9], *Vitis vinifera* (117 members) [7], *Populus trichocarpa* (192 members) [10] and *Zea mays* (more than 200 members) [8]. The members of the *R2R3MYB* family from *Arabidopsis* have been divided into 25 subgroups by Dubos et al. [1]. Comparative phylogenetic studies have identified new *R2R3MYB* subgroups in other plant species for which there are no representatives in *Arabidopsis* (e.g. in *Populus* and *Vitis*), which suggested that these proteins might have specialized roles which have been either lost in *Arabidopsis* or were acquired after divergence from the last common ancestor [7], [10]. The expansion of the *R2R3MYB* gene family in plants fits well with the observation that many (if not all) *R2R3MYB* transcription factors play central roles in plant-specific processes [1].

A growing body of evidence suggests that *R2R3MYB* transcription factors are involved in many significant physiological and biochemical processes [1], [9], [10], such as the regulation of secondary metabolism [5], [11]–[15], the fate of epidermal cells [14], [16]–[19], the control of the cell cycle [20], [21], anther and pollen development [22], [23], axillary meristem formation [24], [25] and participate in plant defense and response to various biotic and abiotic stresses [4], [26]–[30]. *R2R3MYB* family members have also demonstrated roles in regulating plant responses to phytohormonal cues, including abscisic acid [31], [32] and gibberellins [33], and to environmental signals, such as light [34], [35] and water availability [36], [37].

Extensive studies of the *R2R3MYB* gene family in various plant species have provided a better understanding of this gene family. However, little is known about this gene family in cucumber (*Cucumis sativus* L.). To date, none of the *R2R3MYB* genes have been reported in cucumber. Cucumber is not only one of the most important vegetables all over the world, but is also a model system for studies on sex determination and plant vascular biology [38]. Furthermore, its growth and production are severely affected by some abiotic stresses, such as high salinity [39], [40], drought [41], and low temperature [42], [43]. Therefore, the identification and functional study of cucumber stress responses and tolerance genes may elucidate the molecular mechanisms behind the plant stress responses and tolerance and could ultimately lead to improvements in stress tolerance.

A draft of the *Cucumis sativus* genome sequence was reported recently [44]. The genome-wide of *R2R3MYB* genes can now be identified and described. In the present study, genome sequence was searched so that the *CsR2R3MYB* genes could be identified in order to predict protein domain architectures and to assess the extent of conservation and divergence in the cucumber *R2R3MYB* family. A phylogenetic tree combining *Arabidopsis*, *Vitis*, *Oryza*, *Populus* and *Glycine* R2R3MYB proteins was constructed so that their evolutionary relationships and the putative functions of cucumber R2R3MYB proteins could be examined based on *Arabidopsis* R2R3MYB proteins with known functions. Alternative splicing (AS) analysis indicated that 8 out of 55 (∼ 14.54%) cucumber *R2R3MYB* genes underwent AS events, producing a variety of transcripts from a single gene. Tissue-specific analysis was performed and abiotic condition response expression profiles were generated so that genes, which could be potentially participate in the stress signal transduction pathway in cucumber, could be identified. This extended analysis is the first comprehensive study of the *R2R3MYB* gene family in cucumber and provides valuable information for further exploration into the functions of this significant gene family in cucumber. In addition, these results provide information about the relationship between evolution and functional divergence in the *R2R3MYB* family.

## Results

### Identification and Sequence Conservation of Cucumber *R2R3MYB* Genes

One hundred and twenty-six *Arabidopsis* R2R3MYB proteins and the consensus protein sequences of the MYB-binding domain, Hidden Markov Model (HMM) profile (PF00249), were employed as a query to search against the cucumber genome database (http://cucumber.genomics.org.cn/page/cucumber/index.jsp) using the BlastP program. A total of 71 genes in the cucumber genome were identified as possible members of the *CsR2R3MYB* family. To confirm putative *R2R3MYB* genes in the cucumber genome, the amino acid sequences of all 71 proteins were searched for the presence of the R2R3 domain by Pfam and SMART. Following an extensive search for *R2R3MYB* genes, 55 typical *R2R3MYB* genes (named *CsMYB0* to *CsMYB54*) were confirmed from the original data. These 55 cucumber *R2R3MYB* genes were subjected to further analyses (Table 1).

**Table 1:** *R2R3MYB* genes in cucumber.

				Protein	ORF
CsMYB	Gene	Chromosome	Location	Length	Length
				(aa)	(bp)
0	Csa012797	5	16240983–16241855	260	783
1	Csa001207	2	21390922–21391827	301	906
2	Csa001544	1	17395322–17396637	370	1113
3	Csa001869	7	14168602–14169999	280	843
4	Csa002447	3	27266343–27267639	316	951
5	Csa002643	3	27532770–27534415	294	885
6	Csa002717	3	28547965–28549234	233	702
7	Csa003349	7	2571315–2572816	337	1014
8	Csa003351	7	2589707–2591872	271	816
9	Csa003581	2	16162499–16163227	242	729
10	Csa003827	1	5407107–5408283	324	975
11	Csa004520	1	3103403–3103876	157	474
12	Csa004708	3	25039759–25041807	312	939
13	Csa005181	1	1276693–1278035	367	1104
14	Csa005219	1	642044–643354	360	1083
15	Csa005383	1	1449501–1450571	330	993
16	Csa007739	6	7410862–7411826	243	732
17	Csa008131	5	6609315–6610872	300	903
18	Csa008771	3	13015119–13015778	219	660
19	Csa008970	6	2787714–2789069	279	840
20	Csa009054	2	7218688–7220197	355	1068
21	Csa009102	2	6270188–6273644	193	582
22	Csa009345	4	21473897–21474920	280	843
23	Csa009412	4	20666568–20667257	229	690
24	Csa009484	4	21120591–21121382	263	792
25	Csa009573	5	28330408–28331504	294	885
26	Csa009688	5	26872755–26875065	324	975
27	Csa009714	5	27183101–27184341	202	609
28	Csa010143	6	21377201–21378106	301	906
29	Csa011496	2	15634540–15635412	290	873
30	Csa011529	2	15261712–15263031	209	630
31	Csa012498	5	878496–879483	255	768
32	Csa012824	5	15788398–15790543	323	972
33	Csa014650	2	10771778–10773637	229	690
34	Csa015156	6	9033339–9034405	285	858
35	Csa015272	Scaffold000067	573478–575686	267	804
36	Csa016676	4	8888095–8889391	314	945
37	Csa016768	2	12203260–12204149	256	771
38	Csa017164	1	8338540–8340339	318	957
39	Csa017450	3	28805009–28806330	313	942
40	Csa017539	5	16598928–16600943	258	777
41	Csa017970	4	11164743–11166675	377	1134
42	Csa018176	2	22052618–22054320	308	927
43	Csa018350	5	24309660–24310672	267	804
44	Csa018538	2	13984000–13985298	253	762
45	Csa019830	3	3487890–3490183	519	1560
46	Csa022057	Scaffold000270	9766–10899	305	918
47	Csa024079	Scaffold001107	742–1807	241	726
48	Csa013585	7	3801132–3802328	254	765
49	Csa008474	6	5686522–5688185	301	906
50	Csa000156	3	8586660–8587151	105	318
51	Csa020800	6	13006650–13013649	272	819
52	Csa018305	5	3136912–3137766	284	855
53	Csa000784	6	26665502–26666371	289	870
54	Csa019344	7	11844745–11846161	254	765

**Figure 1:**
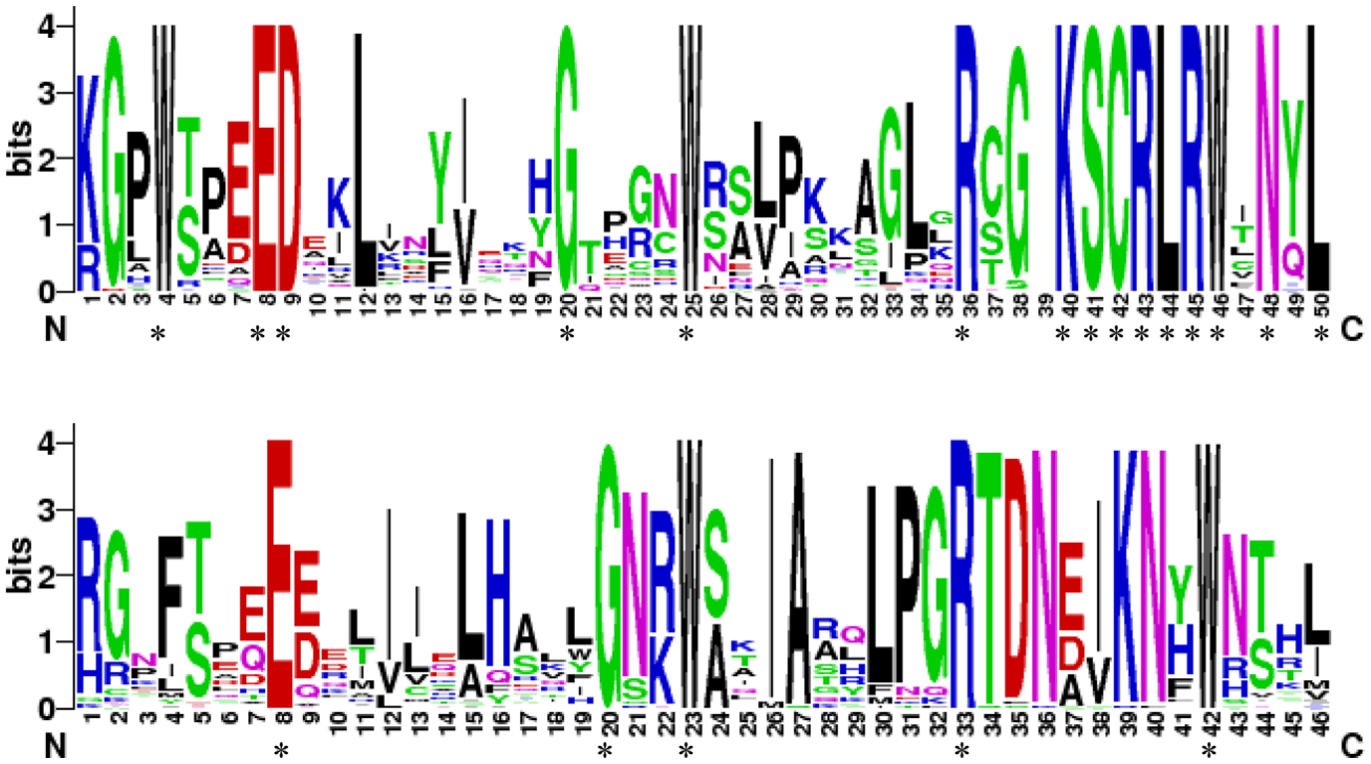
The R2 and R3 MYB repeats are highly conserved across all *CsR2R3MYB* proteins. The sequence logos of the R2 (Top) and R3 (Bottom) MYB repeats are based on full-length alignments of all CsR2R3MYB proteins. The overall height of each stack indicates the conservation of the sequence at that position, whereas the height of letters within each stack represents the relative frequency of the corresponding amino acid. The asterisk indicates the position of the conserved amino acid that is identical among all the 55 cucumber R2R3MYB proteins.

To gain insight into the cucumber R2R3MYB binding domains, sequence logos were produced to examine how well conserved the R2 and R3 repeats were in the R2R3MYB proteins within each residue position. As shown in Fig.1, fifteen and five conserved amino acid residues were identical among the members detected in the R2 and R3 MYB repeat regions, respectively. Within the 55 cucumber R2R3MYB proteins, all the R2 repeat sequences contained three tryptophan residues. However, in the R3 repeats, the first tryptophan residue was replaced by phenylalanine. The second and third tryptophan residues were conserved in all the members. These results were consistent with those from *Arabidopsis* [5], *Populus* [10] and *Triticum* [4].

### Phylogenetic Analysis of the Cucumber *R2R3MYB* Family

**Figure 2:**
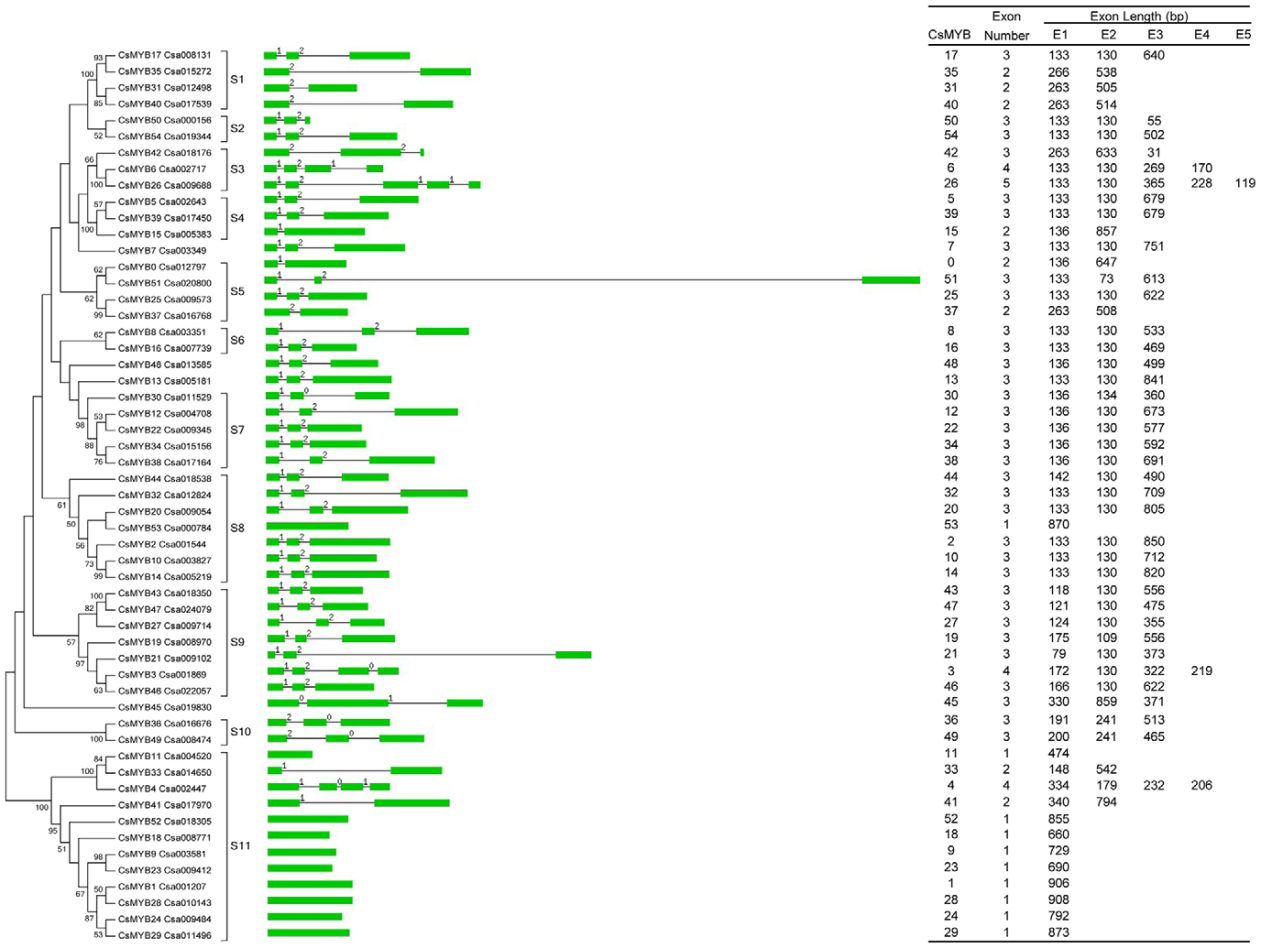
Neighbor-joining (NJ) phylogenetic tree and intron-exon structures of *CsR2R3MYB* family genes. The unrooted phylogenetic tree (the part of left side) from the R2R3MYB domain was depicted by the MEGA 4.0 program with the NJ method. The tree shows the 11 phylogenetic subgroups (S1–S11) with high bootstrap value. The bootstrap values lower than 50 are not shown in the phylogenetic tree. All of 55 gene’s intron-exon structures are described in the middle part. Exons and introns are indicated by green boxes and single lines, respectively. Introns phases 0, 1 and 2 are indicated by numbers 0, 1 and 2, respectively. The length of each *CsR2R3MYB* gene can be estimated using the scale at the bottom. The exon number and length of each gene are listed in the table at right.

The phylogenetic relationship between the CsR2R3MYB proteins was examined by multiple sequence alignment of their MYB binding domain with bootstrap analysis (1,000 replicates). The 55 members of the *CsR2R3MYB* family were subdivided into 11 subgroups, designated S1 to S11, according to clades with at least 50% bootstrap support. Nineteen gene pairs were formed with strong bootstrap support. To compare the two phylogenetic trees on the basis of cucumber R2R3MYB domains and complete protein sequences, respectively, similar subgroups were analyzed, though the classifications of only a few members varied (Fig. 2; Fig. S1). This indicated that the conserved *R2R3MYB* domain was an important unit in CsR2R3MYB protein and the dramatic divergence of the C-terminal regions did not appear to have a large influence on the regulatory function of the corresponding proteins [8].

**Figure 3:**
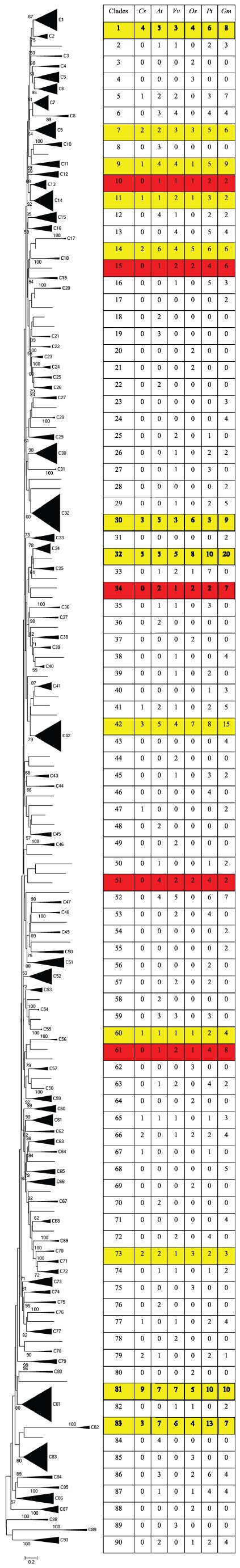
Phylogenetic relationships and subgroup designations in R2R3MYB proteins from cucumber (*Cs*), *Arabidopsis* (*At*), grape (*Vv*), rice (*Os*), poplar (*Pt*) and Soybean (*Gm*). The neighbor-joining tree includes 55 R2R3MYB proteins from cucumber, 126 from *Arabidopsis*, 117 from *Vitis*, 102 from rice, 197 from poplar and 244 from soybean. The bootstrap values lower than 50 are not shown in the phylogenetic tree. The proteins are clustered into 90 subgroups (triangles), designated with a subgroup number (e.g. C1). Forty eight proteins did not fit well into subgroups (lines). The membership of each subgroup is described in the table at right. Several subgroups are highlighted. 12 subgroups (yellow) are shared in all the 6 species. 5 subgroups (red) are shared among other 5 speices but not with cucumber. The uncompressed tree with full taxa names is available as Figure S2.

To obtain information about the evolutionary relationship of the *CsR2R3MYB* genes, an unrooted NJ phylogenetic tree using bootstrap analysis (1000 replicates) was built from alignments of the R2R3MYB complete protein sequences from 55 *CsR2R3MYB*, 126 *AtR2R3MYB*, 117 *VvR2R3MYB*, 102 *OsR2R3MYB*, 197 *PtR2R3MYB* and 244 *GmR2R3MYB* genes (Fig. 3; Fig. S2). The phylogeny was very similar to a previously published phylogeny that included all known *Arabidopsis*, *Vitis*, *Oryza*, *Populus* and *Glycine* R2R3MYB proteins [1], [7], [10], [45]. The resulting tree generated 90 subgroups (triangles), which were designated with a subgroup number (C1–C90). However, 48 proteins did not fit well into any subgroups (lines) (Fig. 3; Fig. S2). The 48 proteins were considered orphans, most likely representing highly diverged lineage-specific R2R3MYB protein sequences.

Phylogenetic analysis of the predicted R2R3MYB protein sequences revealed that there was not equal representation of cucumber, *Arabidopsis*, *Vitis*, *Oryza*, *Populus* and *Glycine* R2R3MYB proteins within the given subgroups (Fig. 3; Fig. S2). Twelve (C1, 7, 9, 11, 14, 30, 32, 42, 60, 73, 81 and 83) were shared in all the 6 species. Among them, phylogeny subgroup C81 included 7AtR2R3MYB, 7VvR2R3MYB, 5 OsR2R3MYB, 10 PtR2R3MYB, 10 GmR2R3MYB and 9 CsR2R3MYB proteins, which suggested that this is an expanded subgroup in cucumber compared with the *Arabidopsis*, grape and rice *R2R3MYB* families but not poplar and soybean.

Seventy subgroups (C2–4, 6, 8, 10, 12, 13, 15–29, 31, 33–40, 43–46, 48–59, 61–64, 68–72, 74–76, 78, 80, 82 and 84–90) were absent in the cucumber genome. Of the 70 subgroups, five ones (C10, 15, 34, 51, 61) were shared among *Arabidopsis*, grape, rice, poplar and soybean but not in cucumber, which suggested that these R2R3MYB proteins may have specialized roles that were acquired or expanded in *Arabidopsis*, grape, rice, poplar and soybean after divergence from the last common ancestor with cucumber. Meanwhile, some species-specific subgroups were also observed, indicating that *R2R3MYB* genes may have evolved or been lost in a single species, following divergence. For example, 10 subgroups (C8, 18, 19, 22, 36, 48, 58, 70, 76 and 84) only contained *Arabidopsis* members, 4 subgroups (C44, 49, 78 and 89) only grape members, 12 subgroups (C3, 4, 20, 21, 37, 62, 64, 69, 75, 80, 85 and 88) only rice members, 2 subgroups (C46 and 56) only poplar members and 10 subgroups (C17, 23, 24, 28, 31, 43, 54, 55, 68 and 71) only soybean members, which indicated that these genes may have special functions in *Arabidopsis*, grape, rice, poplar and soybean, respectively. Interestingly, C66 did not include any *Arabidopsis* R2R3MYB proteins but only members from cucumber, grape, poplar and soybean. This suggested that the genes in C66 may have been lost in *Arabidopsis* during the evolutionary process. The similar reason could also explain that three subgroups (C5, 41 and 65) were absent in the rice genome but not cucumber, *Arabidopsis*, grape, poplar and soybean, Some cucumber R2R3MYB proteins were clustered into *Arabidopsis* functional clades (Fig. S2), which provided an excellent reference to explore the functions of the cucumber *R2R3MYB* genes. For example, CsMYB21 grouped together with *Arabidopsis* AtMYB21 and AtMYB24 into clade 41, referring to control anther development [46], [47]. CsMYB36 and CsMYB49 were clustered into clade 79 and shared a high level of sequence similarity with male gamete cell formation protein AtMYB125 (DUO1). This implied that the possible functions of CsMYB36 and 49 were related to male gamete cell division and differentiation [22]. CsMYB6 and CsMYB26 was grouped into clade 7 with two *Arabidopsis* proteins, AtMYB16 (MIXTA), proposed to control the shape of petal epidermal cells [48] and AtMYB106 (NOK), a negative regulator of trichome branching [49]. This represented a functional clade containing proteins responsible for cell development or morphogenesis. Remarkably, CsMYB 0, 8, 16, 27, 48, 50, 51, 53 and 54 did not fit well into any of the clades, which indicated that the 9 proteins might have specialized roles in cucumber or were acquired after divergence from the last common ancestor with other 5 species.

### Intron–exon Structure of the Cucumber *R2R3MYB* Family

**Figure 4:**
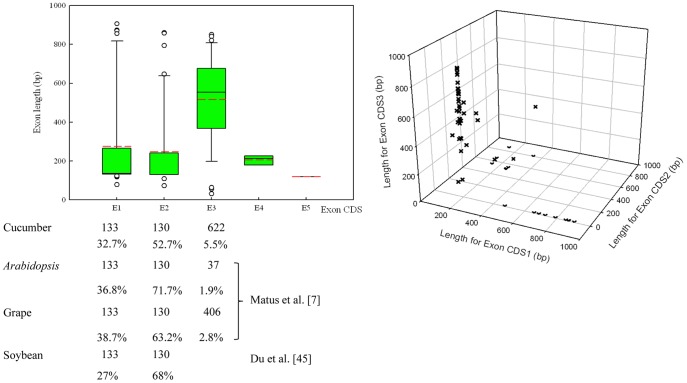
Exon length distribution analysis of the cucumber *R2R3MYB* genes. (A) Exon length values were analysed using Boxplot depicted by SigmaPlot 10.0. Each box represents the exon size range in which 50% of the values for a particular exon are grouped. The mean value is shown as a dotted line (red) and the median as a continuous line. Only four genes possess four exons while one gene was predicted to have five. (B) First, second and third exon lengths distribution of cucumber *R2R3MYB* genes using 3D Scatter Plot depicted by SigmaPlot 10.0.

According to the results of intron–exon structure identification (Fig. 2), within the 55 *CsR2R3MYB* genes, the number of exons ranged from one to five and 44 out of 55 had more than one exon. As shown in Fig. 4A, exon 1 and 2 appeared to be the more restricted in length, while exon 3 was more variable (31–850 bp). Presence of a fourth and fifth exon was exclusive to some specific genes. Despite this variability, the lengths of the first two exons were very similar (exon 1, 133 bp; exon 2, 130 bp) and highly conserved (exon 1, 32.7% occurrence; exon 2, 52.7% occurrence). Although exon 3 was the most diverse in size, *R2R3MYB* families from cucumber, *Arabidopsis* [7], grape [7] and soybean [45] species were similarly distributed when the first three exon lengths were considered (Fig. 4B).

**Figure 5:**
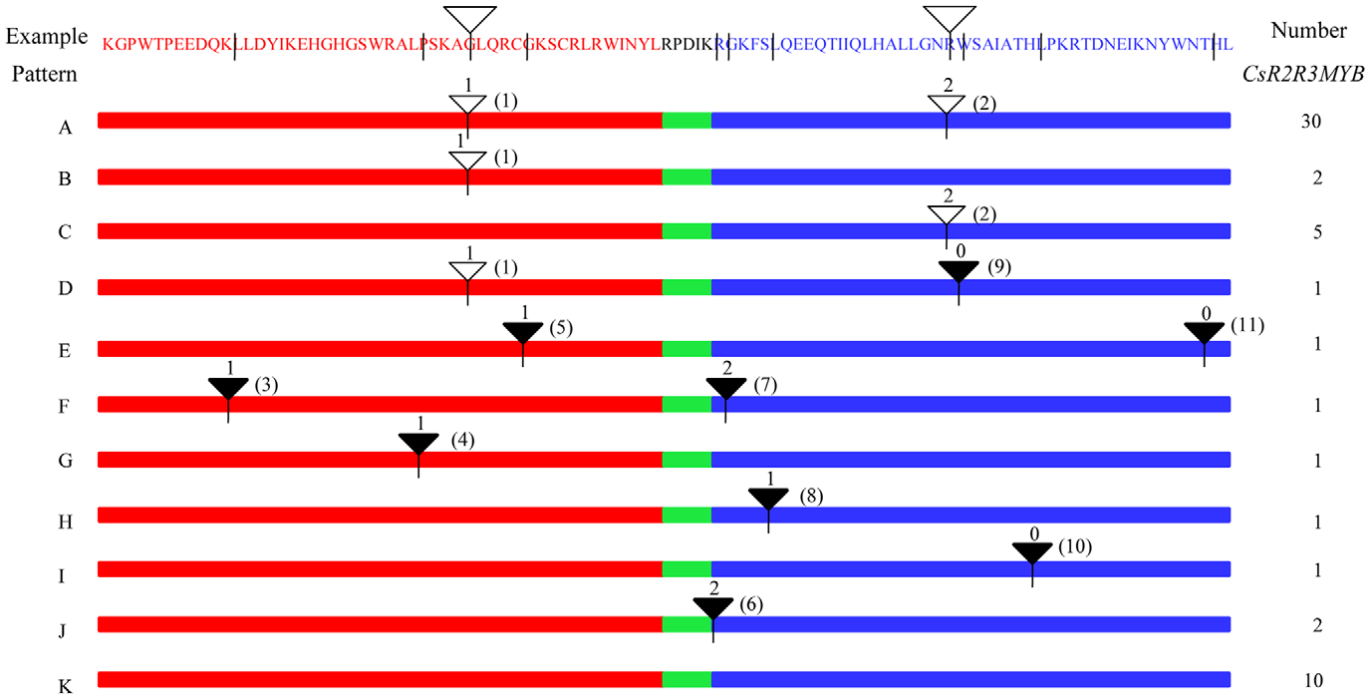
Intron distribution patterns of 55 cucumber *R2R3MYB* genes. Red, blue and green rectangles represented the R2 domain, R3 domain and five amino acids between the R2 and R3 domain, respectively. The intron splicing patterns were designated A-K according to relative positions and phases within the MYB binding domains of the CsR2R3MYB proteins. The white triangles are used when the position of the intron coincides with the intron 1 and intron 2 in example. The black triangles indicate that the location of the intron within the MYB binding domain corresponding to the example (line) is different from the intron1 and intron 2. The numbers above the triangles indicate the splicing phases, 0, 1, 2 refers to phase 0, 1, 2. The markers 1 to 11 beside the triangles show different positions of the introns. The number of cucumber R2R3MYB proteins with each pattern is given at right. Here the position of introns in the variable region has been adjusted manually to make them more contracted.

When the *CsR2R3MYB* gene structures were analyzed further, the number of introns contained in their R2 and R3 domains was determined. All 55 genes, according to relative positions and phases, could be arranged into 11 different splicing patterns (A-K) (Fig. 5). Patterns A to C, composed of one or two intron (s) distributed at two highly conserved specific positions (indicated by white inverted triangles), accounting for approximately 67% of *CsR2R3MYB* genes. Patterns F-I had introns at varying positions in the R2 or R3 domain and were observed in only 11% of the 55 genes. Approximately 22% of these 55 genes (patterns J and K) had no introns at the MYB binding domain. It was noteworthy that two genes (*CsMYB36* and *CsMYB49*) from pattern J had one intron between the R2 and R3 domain and were classified into the same subgroup shown in Fig. 2.

Intron phases with respect to codons were investigated. An intron was designated as occurring in one of three phases. In phase 1, splicing occurred after the first nucleotide of the codon; in phase 2, splicing occurred after the second nucleotide and in phase 0, splicing occurred after the third nucleotide of the codon [50], [51]. Fig. 5 showed that the introns 1 and 2 at the two conserved positions (indicated by white inverted triangles) had phases 1 and 2, respectively. The other introns (intron 3, 4, 5, 6, 7, 8, 9, 10 and 11), with less conserved positions (black inverted triangles), were in phases 0, 1 or 2. Interestingly, all of the introns (1, 3, 4 and 5) in the R2 domain were in phase 1. In contrast, the phases that contained five introns (7, 8, 9, 10 and 11), which were located in the R3 domain, were more variable. This suggested that the splicing phase was highly conserved during the evolution of *CsR2R3MYB* genes. Such conserved splicing patterns and phases were also observed in the MYB gene families of *Arabidopsis* [52], rice [52] and soybean [45].

### Genome Distribution and Gene Duplication of Cucumber *R2R3MYB* Genes

**Figure 6:**
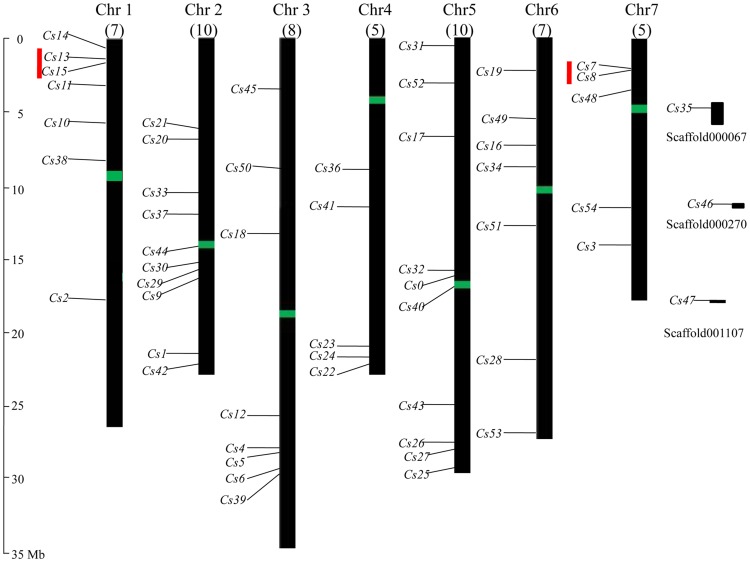
Chromosomal locations and predicted cluster for *CsR2R3MYB* genes. Chromosomal positions of the *CsR2R3MYB* genes are indicated by *CsR2R3MYB* number (*Cs* represented *CsR2R3MYB* assigned in Table 1). The scale is in megabases (Mb). The green bars in the middle of the 7 chromosomes show the rough position of the centromeres. The numbers below the name of the chromosome show the number of *CsR2R3MYB* genes in this chromosome. Three genes could not be localized on a specific chromosome. Red line indicates a gene cluster.

To determine the genomic distribution of the *CsR2R3MYB* genes, the DNA sequence of each *CsR2R3MYB* gene was used to search the cucumber genome database using BLASTN. A total of 52 *R2R3MYB* genes could be mapped on chromosomes 1 to 7 (Table 1; Fig. 6). Three genes (*Csa015272*, *Csa022057* and *Csa024079*) could not be conclusively mapped on any chromosome. Although each of the seven cucumber chromosomes contained some *CsR2R3MYB* genes, the distribution seemed to be uneven (Fig. 6). The largest number of *R2R3MYB* genes were found on chromosomes 2 and 5 (ten genes each), followed by chromosome 3 (eight genes). Seven genes were distributed on each of chromosomes 1and 6. Only five genes were located on each of chromosomes 4 and 7. This analysis revealed that cucumber *R2R3MYB* genes were found in all chromosomes. Relatively high densities of *CsR2R3MYB* genes were found in some chromosomal regions, such as the short arm of chromosome 1, 2 and 3, and the long arm of chromosome 4. In contrast, several large chromosomal regions lacked *CsR2R3MYB* genes, such as the top of chromosome 2 and 4, the bottom of chromosome 1 and the central sections of chromosome 1, 3 and 7.

In this study, gene duplication events, including tandem and segmental duplications, were investigated with the purpose of elucidating the mechanism behind the expansion of the *CsR2R3MYB* gene family that is thought to have occurred during the evolutionary process [53]–[55]. Huang et al. [44] reported that the recent whole-genome duplication event was absent in the cucumber genome. However, a few tandem duplications have been shown to exist in cucumber. The phylogenetic analysis results indicated that there were no tandem duplicated genes in the *CsR2R3MYB* family because no cucumber paralogs could be detected, which indicated the absence of a recent tandem duplication event in the *CsR2R3MYB* family. The method utilized by Schauser et al. [56] was used to detect whether or not segmental duplication events had occurred in the *CsR2R3MYB* family and found that no *CsR2R3MYB* genes could be attributed to segmental duplication. Similar results were also found in the cucumber *WRKY* [57], *MADS* [58], *LOX* [59] and *ERF* [60] families.

According to Holub’s [61] description, a chromosome region containing two or more genes within 200 kb can be defined as a gene cluster. Analysis of the positions of the 55 *CsR2R3MYB* genes in the cucumber genome did not reveal a strong clustering on particular chromosomes (Table 1; Fig.6). The exceptions were *CsMYB 7* and *8*, *CsMYB 13* and *15*, which were located within 17 kb on chromosome 7and171 kb of each other on chromosome 1, respectively. Since none of the neighboring genes were duplicated, the two clusters likely arose from a local rather than a whole genome duplication event.

### Alternative Splicing (AS) Analysis

Alternative splicing (AS) is the mechanism by which a common precursor mRNA produce different mRNA variants, by extending, shortening, skipping, or including exon sequences, or retaining intron sequences [45]. The combinatorial joining of exons by AS is an elegant mechanism that most eukaryotes use to generate several distinct proteins from a single transcript [62]. In this paper, PCR amplification to screen possible AS in all 55 *CsR2R3MYB* genes were conducted, and several distinctively spliced transcripts were successfully obtained.

**Figure 7:**
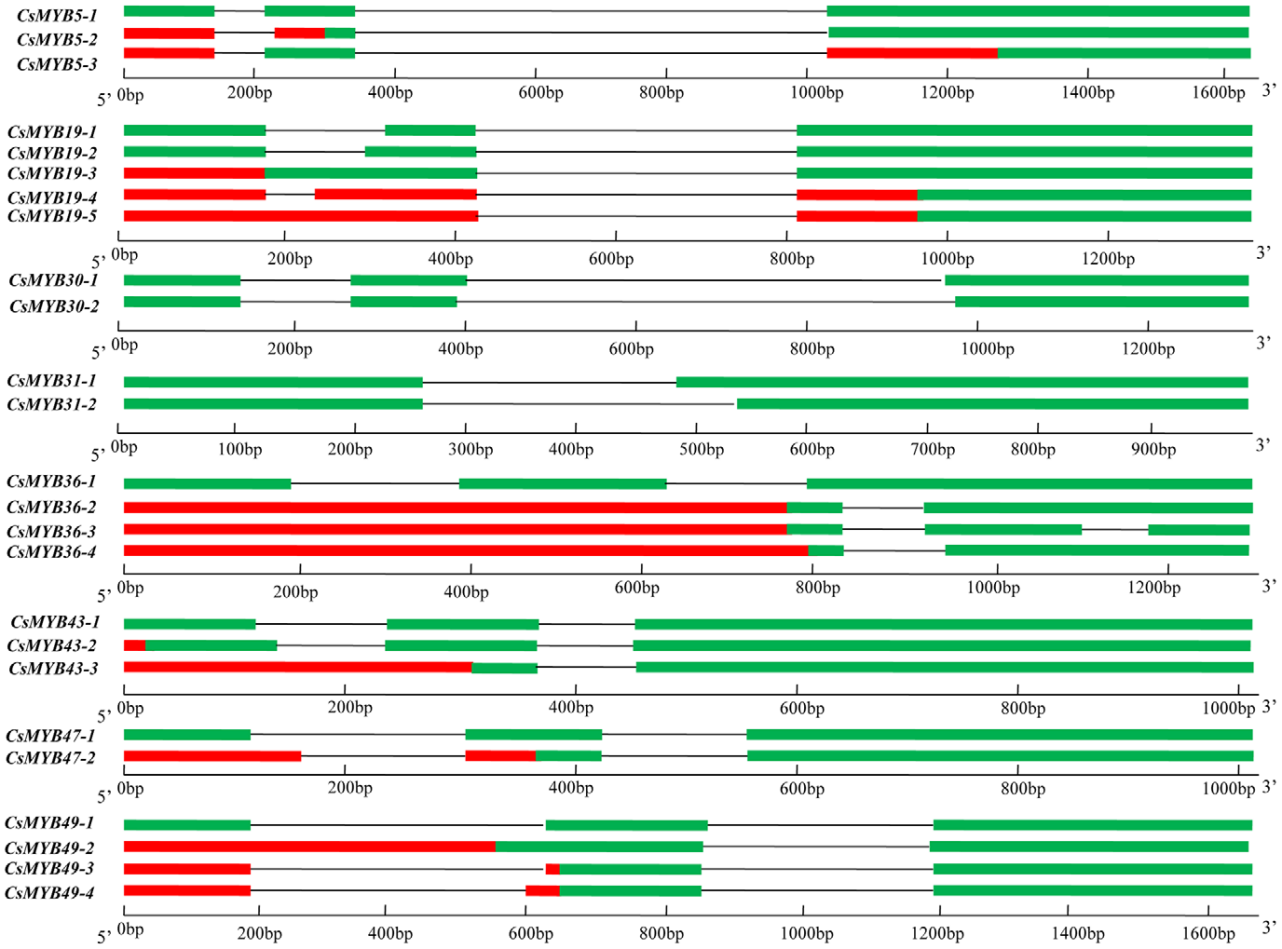
Schematic representation of the differently spliced transcripts of *CsMYB5*, *19*, *30*, *31*, *36*, *43*, *47* and *49*. Green boxes, red boxes and lines are shown as putative open reading frames (ORFs), upstream ORFs and introns, respectively.

As shown in Fig. 7, 8 of 55 *R2R3MYB* genes in cucumber contain two to five alternative structures that indicate they had undergone AS, producing a variety of transcripts from a single gene. Two distinctively spliced transcripts were found for *CsMYB30*, 31 and *47*, three for *CsMYB5* and *43*, four for *CsMYB36* and *49*, and five for *CsMYB19*, respectively. In general, these AS events resulted in a variety of sequence insertions and/or deletions in the corresponding ORFs. For instance, a 21bp AS site in R2 repeat of *CsMYB19-2* allowed the lengthening of 16 and 7 amino acids, respectively. However, a 15 bp and 57 bp AS sites in *CsMYB30* and *31* resulted in a deletion of 5 and 19 amino acids in R3 repeat, respectively. Interestingly, we observed that some of the AS events changed the type of R2R3MYB protein. For example, A 189 bp AS site of *CsMYB5* resulted in a frame shift, which changed the R2R3MYB (*CsMYB5-1*) into a single-repeat MYB type (*CsMYB5-2*). Similarly, *CsMYB19-3*, *CsMYB30-2, CsMYB43-2*, *CsMYB47-2*, *CsMYB49-2*, *-3* and *-4* were also confirmed as single-repeat MYB genes. In contrast, although AS in *CsMYB19-2* resulted in an insertion of 21bp in R2 repeat and a deletion of 57bp in R3 repeat of *CsMYB31-2*, they were still typical *R2R3MYB* genes. Remarkably, some alternative types of splicing resulted in a long deletion at the 5′ terminus, for example, *CsMYB5-3*, *CsMYB19-4*, *-5* and *CsMYB36-2*, *-3*, *-4*. However, these transcripts were unlikely to code a protein. The reasons were as follows: the seven upstream ORFs existing in the long leader region (at least 515 bp) of these transcripts would strongly repress translation of the downstream ORF [62]–[66]; and it has been shown that the transcripts with long, AUG-burdened leader sequences were incapable of supporting protein synthesis [67]–[69]. More interestingly, all ORFs encode proteins that differ only in the MYB domains at the 5′ terminus.

**Figure 8:**
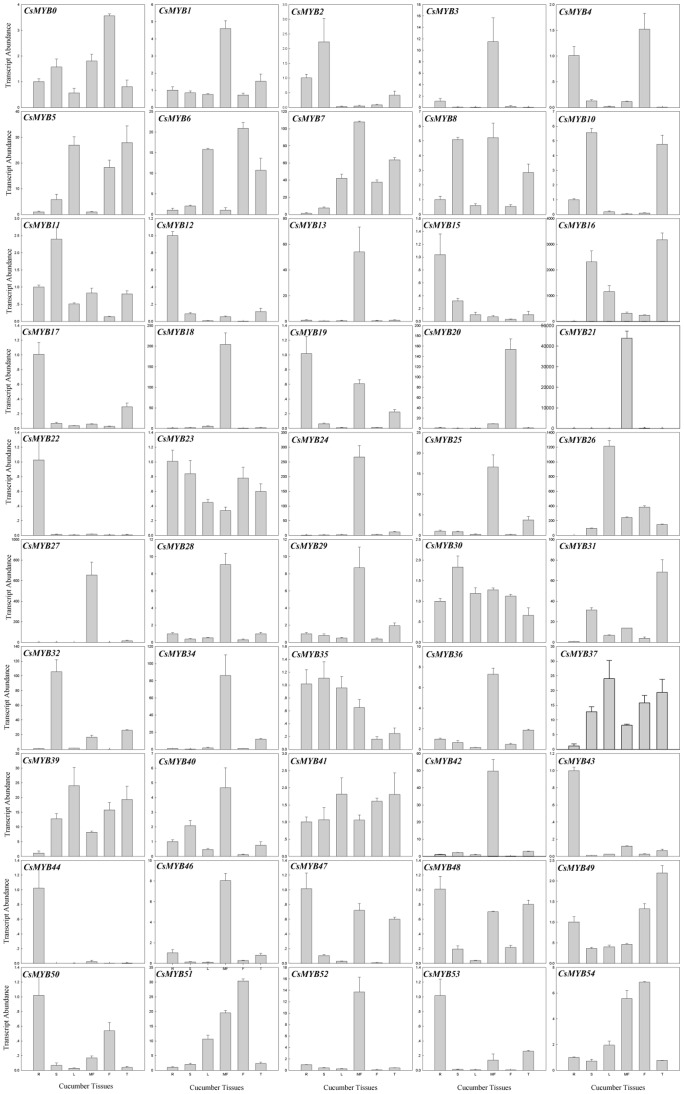
Tissue-specific expression profiles of 50 cucumber *R2R3MYB* genes. Relative transcript abundances of *CsR2R3MYB* genes were examined by qRT-PCR. The Y axis is the scale of the relative transcript abundance level. The X axis is the tissues of cucumber. Total RNA was isolated from roots (R), stems (S), leaves (L), male flowers (MF), fruits (F) and tendrils (T), respectively. The cucumber *β-actin* gene (GenBank AB010922) was performed as an internal control. Five genes (*CsMYB9*, *CsMYB14*, *CsMYB33*, *CsMYB38* and *CsMYB45*) showed very low expression in the above tissues, so the qRT-PCR results of these five genes were not displayed. The PCR primers were designed to avoid the conserved region and to amplify products of 150 to 300 bp. Primer sequences were shown in detail in Table S2.

As Li et al. [62] reported that *AtMYB59* and *AtMYB48* underwent similar AS events, moreover, the conserved AS pattern was also found in two rice homologous genes (*Os11g47460* and *Os12g37970*). As shown in Fig. 3 and Fig. S2, *CsMYB43* and *CsMYB47* were two homologous genes of *AtMYB59*, *AtMYB48*, *Os11g47460* and *Os12g37970* in cucumber. The results in Fig. 7 demonstrated that these two cucumber homologous genes undergo similar AS with *AtMYB59*, *AtMYB48*, *Os11g47460* and *Os12g37970*.

### Expression Profiles for Cucumber *R2R3MYB* Genes in Different Tissues and Under Different Abiotic Conditions

Semi and real-time quantitative RT-PCR were both used to detect the expression patterns for all cucumber *R2R3MYB* genes in the roots, stems, leaves, male flowers, fruits and tendrils, and under three treatments. The expression profiles of the 55 cucumber *R2R3MYB* genes showed different patterns of tissue-specific expression (Fig. 8; Fig. S3). Nineteen genes (*CsMYB* 0–2, 5, 6, 10, 11, 23–25, 28, 29, 34, 35, 36, 37, 41, 43 and 49) (34.5%) were expressed in all tissues tested, although the transcript abundance of some genes in spatial tissues was very low. Five genes (*CsMYB9*, *CsMYB14*, *CsMYB33*, *CsMYB38* and *CsMYB45*) showed very low transcript abundances when tested using both semi and real-time quantitative RT-PCR in the above tissues which may be pseudogenes, or may be expressed at specific developmental stages, under special conditions or have higher transcript abundance in other tissues, e.g., seeds. The rest of the genes showed spatial variations in transcript abundance, with high levels of transcript abundance in one or some tissues and low transcript abundance in others. For example, *CsMYB5, CsMYB7, CsMYB16* and *CsMYB26* showed high levels of transcript abundance in stems, leaves, male flowers, fruits and tendrils but low levels in the roots. The transcript abundances of *CsMYB15, CsMYB22, CsMYB43* and *CsMYB47* were higher in the roots than any other tissues. Only two genes, *CsMYB18* and *CsMYB21*, showed tissue-specific expression and were only detected in male flowers. These results indicate that the cucumber *R2R3MYB* genes are involved in various aspects of physiological and developmental processes.

**Figure 9:**
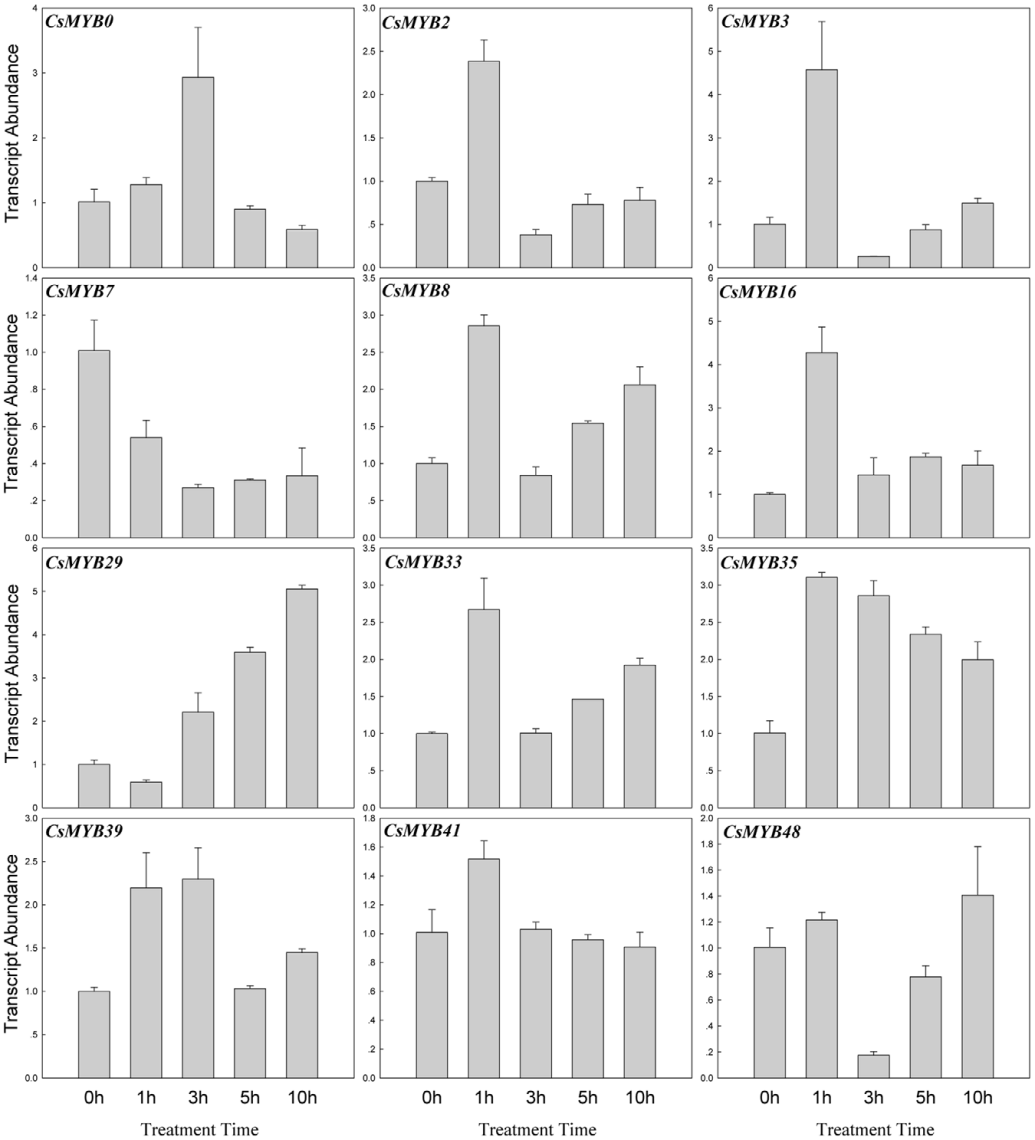
Expression patterns of the 12 cucumber *R2R3MYB* genes under NaCl (100 mM) treatment. Relative transcript abundances of *CsR2R3MYB* genes were examined by qRT-PCR. The Y axis is the scale of the relative transcript abundance level. The X axis is the time course of NaCl treatment. The cucumber *β-actin* gene (GenBank AB010922) was performed as an internal control. The PCR primers were designed to avoid the conserved region and to amplify products of 150 to 300 bp. Primer sequences were shown in detail in Table S2.

**Figure 10:**
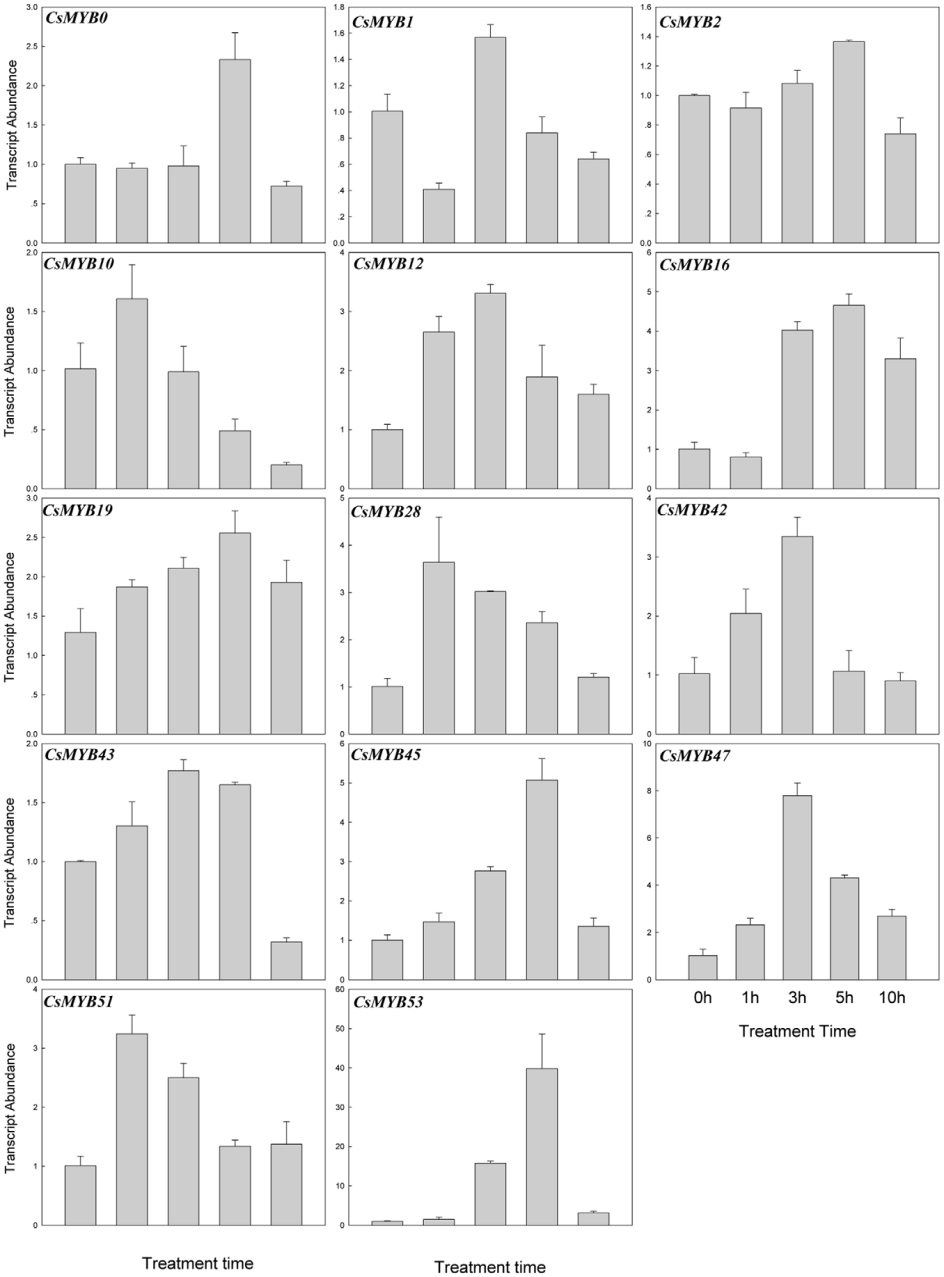
Expression patterns of the 14 cucumber *R2R3MYB* genes under ABA (100 µM) treatment. Relative transcript abundances of *CsR2R3MYB* genes were examined by qRT-PCR. The Y axis is the scale of the relative transcript abundance level. The X axis is the time course of ABA treatment. The cucumber *β-actin* gene (GenBank AB010922) was performed as an internal control. The PCR primers were designed to avoid the conserved region and to amplify products of 150 to 300 bp. Primer sequences were shown in detail in Table S2.

**Figure 11:**
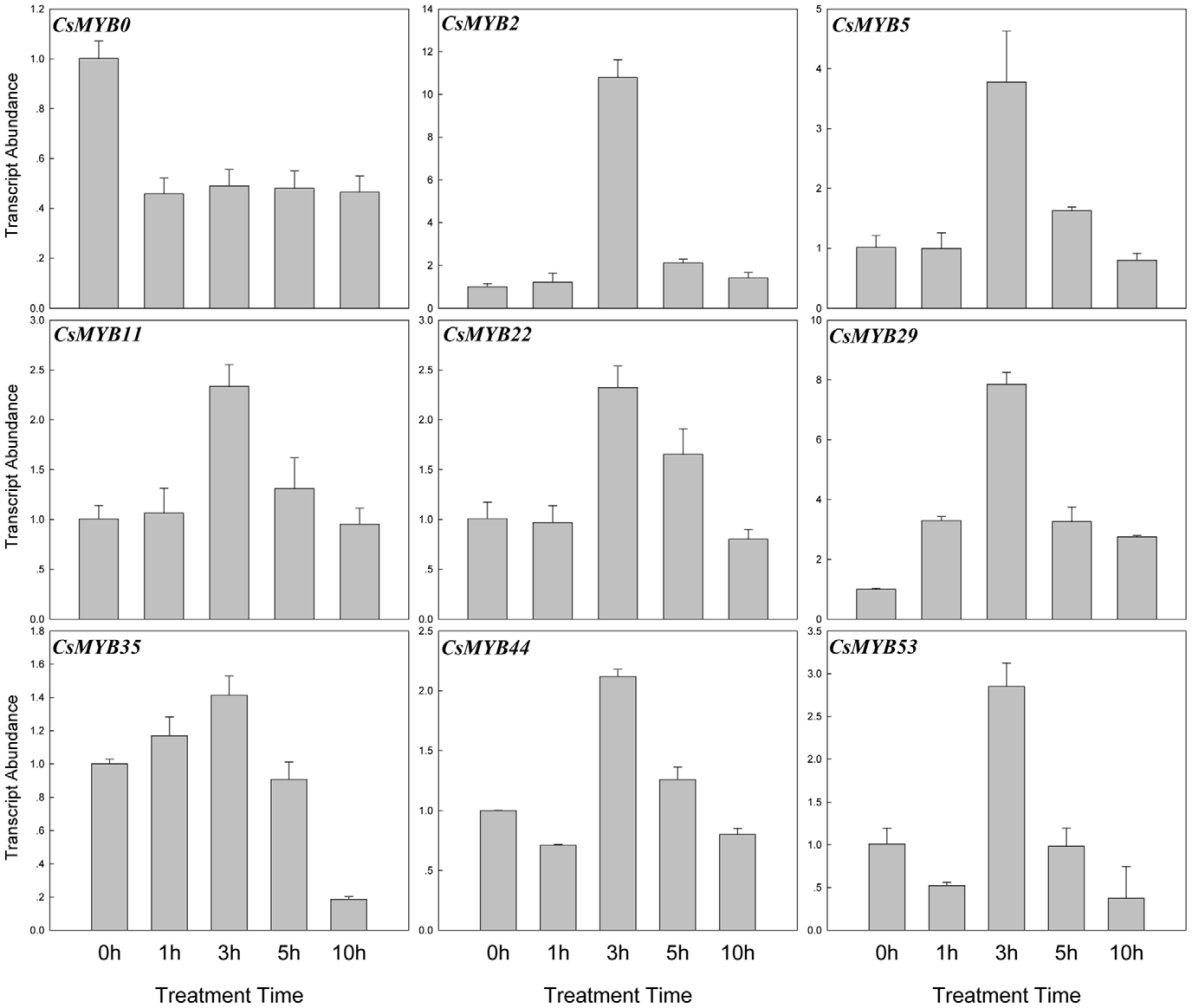
Expression patterns of the 9 cucumber *R2R3MYB* genes under low temperature (4°C) treatment. Relative transcript abundances of *CsR2R3MYB* genes were examined by qRT-PCR. The Y axis is the scale of the relative transcript abundance level. The X axis is the time course of low temperature treatment. The cucumber *β-actin* gene (GenBank AB010922) was performed as an internal control. The PCR primers were designed to avoid the conserved region and to amplify products of 150 to 300 bp. Primer sequences were shown in detail in Table S2.

Mounting evidence suggests that *R2R3MYB* transcription factors play important roles in the response to abiotic stresses [1]. In this study, the transcript abundances of the *CsR2R3MYB* genes at the three-true-leaf stage were investigated under NaCl (100 mM), low temperature (4°C) and ABA (100 µM) treatments. Leaves were harvested after being treated for 0, 1, 3, 5 and 10 h, respectively. The results indicated that 27 (∼49.1%) genes responded to at least one treatment, which included 12 genes responding to NaCl treatment, 14 genes to ABA and 9 genes to low temperature, which suggested that these *CsR2R3MYB* genes were involved in responsive of high salinity, ABA signaling and low temperature, respectively (Fig. 9, 10, 11 and S4). Among these genes, 4 genes (*CsMYB16, 29, 35* and *53*) were able to respond to two treatments and 2 (*CsMYB0* and *2*) genes to all three treatments. The rest 21 genes only responded to a single treatment. The expression of 10, 10 and 7 genes were induced by NaCl, ABA and low temperature treatment, respectively, whereas 2, 4 and 2 genes were repressed, respectively (Fig. 9, 10, 11 and S4; Table S3). Interestingly, some genes behaved in an opposite manner to their expression profile when subjected to different treatments. For example, *CsMYB16* was induced by high salinity but were repressed by ABA, and *CsMYB0* and *53* were induced by ABA but were repressed by low temperature.

## Discussion

### Characterization of the Cucumber *R2R3MYB* Family

R2R3MYBs are widely distributed in higher plants and comprise one of the largest known families of regulatory proteins [8], [10]. However, no related information has been reported in cucumber. This study identified and characterized 55 cucumber *R2R3MYB* genes through genome-wide analysis. Compared to *Arabidopsis* (126) [5], [7], *Vitis* (117) [7], [10], rice (102) [9], poplar (197) [10] and soybean (244) [45], the size of the *R2R3MYB* family was small in cucumber, which suggested that the *R2R3MYB* gene family in *Arabidopsis*, *Vitis*, *Oryza*, *Populus* and *Glycine* had expanded compared to cucumber.

Complete and accurate annotation of genes is an essential starting point for further evolution and function study in gene family. A total of 55 *CsR2R3MYB* genes from 26682 cucumber annotated genes in cucumber genome were identified. Moreover, the draft genome sequence of *Cucumis sativus var. sativus L.*, assembled using a novel combination of traditional Sanger and next-generation Illumina GA sequencing technologies to obtain 72.2-fold genome coverage, and the high coverage of the cucumber genome by this assembly was also confirmed using the available EST, fosmid and BAC sequences [44]. Therefore, the low number of *CsR2R3MYB* family was not the result of inadequate depth of genome coverage.

Many angiosperms underwent whole genome duplication events (γ, β, α). The recent gene duplication events were the most important for the rapid expansion and evolution of gene families [54], [57]. *Arabidopsis* (as well as rice and poplar) underwent the recent duplication events, which led to the large-scale expansion of the *R2R3MYB* family in their genome [54], [70]. However, Huang et al. [44] reported that the cucumber genome was not part of the recent whole-genome duplication events and tandem duplications. The method utilized by Schauser et al. [56] was used to detect whether recent small duplication blocks occurred in the *CsR2R3MYB* family. This study found no *CsR2R3MYB* gene locus on any recent duplication blocks. In addition, phylogenetic analysis revealed that the cucumber *R2R3MYB* family contained nineteen sister pairs. However, none of these pairs were genetically linked to each other on their corresponding chromosomal locations, which indicated the absence of recent tandem duplication event in *CsR2R3MYB* genes. Furthermore, the cucumber genome contained the smallest average gene family size (1.71) compared to *Arabidopsis*, poplar, rice and grape [44]. This may explain, in part, to the small number of genes found in cucumber.

### Phylogenetic Analysis and Evolution of Cucumber *R2R3MYB* Genes

Phylogenetic analysis of the R2R3MYB proteins have been conducted extensively in *Arabidopsis* [1], [5], [7], grape [7], poplar [10], rice [9] and soybean [45], and the evolutionary relationship of this gene family within and among the different species has been systematically studied. To obtain an overall picture of the 55 cucumber R2R3MYB proteins and their relationships with those of *Arabidopsis*, grape, rice, poplar and soybean, phylogenetic trees combining cucumber, *Arabidopsis*, grape, rice, poplar and soybean R2R3MYB proteins were constructed, which divided the 841 R2R3MYB into 90 clades and the 55 CsR2R3MYB members into 20 clades. There are anatomical and physiological differences between cucumber, *Arabidopsis*, grape, rice, poplar and soybean, in addition, the gene loss and lineage-specific expansions were likely to be accounted for by genomic drift [71], so it is possible that some clades could have expanded differently in the cucumber, *Arabidopsis*, grape, rice, poplar and soybean *R2R3MYB* families.

Seventy clades did not include any cucumber R2R3MYB, which suggested that these clades were either lost in cucumber or were acquired after divergence from the last common ancestor. For example, the subgroup of C59 genes is known to be involved in epidermis cell-fate determination in *Arabidopsis*. In cucumber, no C59 subgroup genes were observed, which indicated that the possible gene loss and/or lineage-specific expansions, which may reflect species-specific adaptations [71]. The possible reason could be that multi-cellular trichomes in cucumber (as well as *Solanum lycopersicum*) develop through a transcriptional regulatory network that differs from those regulating unicellular trichome formation in *Arabidopsis* (and perhaps cotton) [72], [73]. The *AtMYB75*, 90, *113* and *114* genes in subgroup C52 play a role in the regulation of anthocyanin biosynthesis [74], [75]. There have been at least five, six and seven C52 subfamily members identified in *Vitis* [7], *Populus* [10] and soybean [45], respectively. It would be interesting to characterize the possible mechanism underlying the absence of anthocyanin-related *R2R3MYB* genes in the cucumber genome. The reason of the absence of epidermis cell-fate determination and anthocyanin-related *R2R3MYB* genes in cucumber perhaps is that these related *R2R3MYB* genes were not identified in this paper. So it is possible that new Cs*R2R3MYB* genes could be identified in the future as annotations improve.

Clade 66 did not include any *Arabidopsis* R2R3MYB and only members from cucumber, grape, rice, poplar and soybean, which implied that these proteins might have specialized roles that were either acquired or expanded in cucumber, grape, rice, poplar and soybean lineages. Similar reasons could explain why none of the rice R2R3MYB members were grouped within clades 5, 41 and 65.

As shown in Fig. 3, several cucumber R2R3MYB proteins were clustered into some *Arabidopsis* functional clades, which provided valuable information on the functions of cucumber *R2R3MYB* genes. Remarkably, none of the cucumber proteins were grouped within the *Arabidopsis* ‘glucosinolate biosynthesis’ clade (C19). A previous study indicated that this clade was derived from a β-type duplication event [76] after *Arabidopsis* diverged from monocots but before diverging from brassicas [77], [78], which may explain the reason for its absence in cucumber, wheat [4], grape [7], rice [7], [9], poplar [10] and soybean [45].

In addition, CsMYB0, 8, 16, 27, 48, 50, 51, 53 and 54 did not fit well into any of the clades, suggesting a gene acquisition mechanism from the most recent common ancestor with other 5 species during the evolution. Our expression analysis revealed that cucumber *R2R3MYBs* had a variety of expression patterns in different tissues. Therefore, we believe that these genes may regulate essential biological processes during cucumber development.

Usually, the pattern of intron positioning can provide important evidence for evolutionary relationships. Previous studies demonstrated that the intron-exon structure was conserved within the same subgroup, but differed between subgroups in the MYB gene family in *Arabidopsis*, rice [52] and soybean [45]. Unexpectedly, among the 11 subgroups, *R2R3MYB* genes in six subgroups (1, 3, 4, 5, 8, and 11) did not always show similar intron-exon structures, respectively. In addition, intron-exon structure was not conserved, even in the same sister pair (*CsMYB17* and *35*; *CsMYB0* and *51*; *CsMYB25* and *37*; *CsMYB20* and *53* and *CsMYB11* and *33*). Furthermore, the intron phases were not conserved within the six subgroups and five sister pairs either. These results combined with gene duplication analysis, suggested that these five gene pairs, as well as the other 14 pairs, were not duplicated genes and confirmed the absence of recent whole-genome duplication events and tandem duplications in the *CsR2R3MYB* family.

As previously observed in *Arabidopsis*, grape [7] and soybean [45] *R2R3MYB* genes, the modal lengths of the first two exons were very similar (exon 1, 133 bp; exon 2, 130 bp) and highly conserved. The exon length of the *CsR2R3MYB* family was also investigated and the results showed that the first two exons lengths were very similar to *Arabidopsis*, grape and soybean, which suggested that MYB binding domains could be partially conserved because exons coding for this domain have all evolved with restricted lengths.

### Alternative Splicing (AS) Analysis

AS of pre-mRNAs is one of the most complex cellular processes in eukaryotes and accounts for a large proportion of proteomic complexity [62], [79], [80]. This allows production of many gene products with enriched functions from a single coding sequence. However, only a small number of AS events have been reported in plants. To date, up to 18 (∼14.29%) *R2R3MYB* genes in *Arabidopsis* underwent AS events [45]. In the present study, AS of *R2R3MYB* genes were detected. We found that 8 of 55 (∼14.54%) *R2R3MYB* genes in cucumber contained two to five alternative structures, which indicated that they had undergone AS, thus producing a variety of transcripts from a single gene (Fig. 7).

Some of the AS events changed the R2R3MYB into a single-repeat MYB type, for example, *CsMYB5-2*, *CsMYB19-3*, *CsMYB30-2*, *CsMYB43-2*, *CsMYB47-2*, *CsMYB49-2*, *-3* and *-4* were confirmed as single-repeat *MYB* genes. As all ORFs encode proteins that differ only in the MYB domains, *CsMYB5*, *19*, *30*, *31*, *36*, *43*, *47* and *49* will be able to encode MYB proteins with one or two MYB repeats, which are known to bind DNA. Therefore, these types of MYB proteins may have binding affinities to different target genes. We also observed that six homologous genes (*AtMYB59*, *AtMYB48*, *Os11g47460*, *Os12g37970*, *CsMYB43* and *CsMYB47*) in C73 [62] underwent similar AS events. This AS pattern, which may have occurred before the divergence of monocots and dicots, was conserved in this subgroup of genes during evolution [62]. A previous study on the MYB genes of *Arabidopsis* and rice indicated that the intron-exon structure was conserved among subgroups [52]. Similar results were also observed in cucumber *R2R3MYB* family. These results demonstrated that besides the conserved intron-exon structure, the AS pattern may also be conserved in some subgroups of MYB genes in both monocotyledonous (rice) and dicotyledonous (*Arabidopsis* and cucumber) plants, although its biological significance is unknown yet [62].

Some alternative types of splicing resulted in a long deletion at the 5′ terminus, for example, *CsMYB5-3*, *CsMYB19-4*, *-5* and *CsMYB36-2*, *-3*, *-4*. Since these seven transcripts were unlikely to encode a protein, the biological relevance of this type of transcript remains to be determined.

### Expression Analysis of Cucumber *R2R3MYB* Genes Response to Abiotic Conditions

Numerous R2R3MYB proteins have been characterized by genetic analysis and have been found to occur in response to various abiotic stresses [1], [4]. However, no *R2R3MYB* family genes have been shown to respond to abiotic conditions in cucumber. For this reason, the expression patterns of cucumber *R2R3MYB* genes were investigated under NaCl (100 mM), low temperature (4°C) and ABA (100 µM) treatment, respectively. The results demonstrated that 27 genes responded to at least one treatment, of which 6 genes responded to multiple treatments. Additionally, some genes showed opposing expression patterns under different stress conditions, such as *CsMYB0*, *CsMYB16* and *CsMYB53*, which indicated that they played a major role in the plant response to abiotic conditions and involved in communication between different signal transduction pathways.

## Materials and Methods

### Database Search and Sequence Conservation Analysis of Cucumber *R2R3MYB* Genes

126 *Arabidopsis* R2R3MYB proteins sequences were obtained from TAIR [1], [5], [7]. 117 *Vitis vinifera* and 197 *Populus trichocarpa R2R3MYB* genes were obtained from Wilkins et al. [10], and the corresponding protein sequences were downloaded from the International Grape Genome Program’s (IGGP) Web site (http://www.genoscope.cns.fr/externe/English/Projets/Projet_ML/projet.html) and Joint Genome Institute P. trichocarpa version 1.1 Web site (http://genome.jgi-psf. org/Poptr1_1/Poptr1_1.home.html), respectively. 102 *Oryza sativa R2R3MYB* genes were obtained from Chen et al. [9] and the corresponding protein sequences were downloaded from the International Grape Genome Program’s (IGGP) Web site (http://rice.plantbiology.msu.edu/analyses_search_locus.shtml). 244 *Glycine max R2R3MYB* genes were obtained from Du et al., [45] and the corresponding protein sequences were downloaded from the Joint Genome Institute (JGI) Glycine max version 7.0 website (http://www.phytozome.net/cgi-bin/gbrowse/soybean/). It is important to note that while Chen et al. [9] identified 109 typical R2R3 MYB proteins, careful scrutiny of the protein sequences revealed that 7 (Os01g11200, Os01g59660 (9629.m05862 and 9629.m05863), Os02g49992, Os08g43450, Os02g49250 and Os04g46390) of these genes were not typical R2R3 MYB proteins and so were excluded from the analysis described here. The cucumber annotated (predicted) genes and proteins were obtained from Cucumber Genome Sequencing Project. This annotated data can be downloaded from Cucumber Genome DataBase (http://cucumber.genomics.org.cn/page/cucumber/index.jsp).

126 Arabidopsis R2R3MYB proteins were used as query sequences and Blastp searches against the predicted cucumber proteins. In addition, the Hidden Markov Model (HMM) profile for the MYB binding domain (PF00249) from the Pfam database (http://pfam.janelia.org) was also applied as a query to identify all MYB containing sequences in cucumber by searching MYB binding domain sequence against the cucumber genome database using BlastP program. To further verify the reliability of these candidate sequences, the Pfam database (http://pfam.sanger.ac.uk/search) and SMART (http://smart.embl-heidelberg.de/) [81] were used to confirm each candidate CsR2R3MYB protein as a member of R2R3MYB family.

To analyze the features of the MYB domain of cucumber R2R3MYB proteins, the sequences of R2 and R3 MYB repeats of 55 CsR2R3MYB proteins were aligned with the ClustalX 1.81 and adjusted manually. The sequence logos for R2 and R3 MYB repeats were obtained by submitting the multiple alignment sequences to the website (http://weblogo.berkeley.edu/logo.cgi) [82].

### Phylogenetic Analysis

Multiple sequence alignments were performed using ClustalX 1.81 with default parameters, and the alignments were then adjusted manually before phylogenetic tree constructed. A phylogenetic tree was constructed with the aligned R2R3MYB binding domain and full predicted protein sequences of 55 *CsR2R3MYB* genes using MEGA 4 [83], respectively. The neighbor-joining (NJ) method was used with the following parameters: poisson correction, pairwise deletion, and bootstrap (1,000 replicates; random seed). The complete amino acid sequences of 841 R2R3MYB proteins, including 126 AtR2R3MYB, 117 VvR2R3MYB, 102 OsR2R3MYB, 197 PtR2R3MYB, 244 GmR2R3MYB and 55 CsR2R3MYB, were used to construct NJ tree using MEGA 4 [83]. Classification of the *CsR2R3MYB* genes was then performed according to their phylogenetic relationships with their corresponding Arabidopsis, grape, rice, poplar and soybean *R2R3MYB* genes.

### Intron-exon Structure Analysis

The DNA and cDNA sequences corresponding to each predicted gene from the cucumber genome and annotation database CuGI were downloaded, and then the intron distribution pattern and splicing phase were analyzed using the web-based bioinformatics tool GSDS (http://gsds.cbi.pku.edu.cn/) [84].

### Genome Distribution and Gene Duplication Analysis

Genes were mapped on chromosomes by identifying their chromosomal position provided in the Cucumber Genome Database. The distribution of *CsR2R3MYB* family members throughout the cucumber genome was drawn manually. To detect the segment duplicated events, the method of Schauser et al. [56] was used. Tandem duplicated genes were identified using the method provided by He et al. [85] and Hu and Liu [58]. Software DNAMAN 5.2.2 was used to analyze the *CsR2R3MYB* homologs in the phylogenetic tree for similarity.

### Alternative Splicing and Expression Analysis

Cucumber (*Cucumis sativus* L. cv. ‘Daqingba’) seeds were germinated on moist filter paper in an incubator at 28°C for 1 day. The germinated seeds were sown into soil mixture in the greenhouse at Shandong Agricultural University. After 10 days, batches of ten seedlings were transferred to a plastic tank filled with an aerated nutrient solution (pH 6.0–6.5) containing: Ca (NO_3_)_2_∶3.5 mM, KNO_3_∶7 mM, KH_2_PO_4_∶0.78 mM, MgSO_4_∶2 mM, H_3_BO_3_∶29.6 µM, MnSO_4_∶10 µM, Fe-EDTA: 50 µM, ZnSO_4_∶1.0 µM, H_2_MoO_4_∶0.05 µM and CuSO4∶0.95 µM [86]. The experiment was carried out in an illuminated incubator and the air temperature (25°C during the day and 18°C during the night) and light intensity (400 µmol m^−2^ s^−1^) regimes were maintained throughout each treatment. When the cucumber seedlings were at the three-true-leaf stage, three treatments were conducted respectively: 100 mM NaCl, 100 µM ABA, 4°C. Leaves for RNA extraction were harvested at 0, 1, 3, 5 and 10 h after the three treatments, respectively. The roots, stems, leaves, male flowers, fruits and tendrils of mature plants were collected separately used for tissue specific expression analysis.

Total RNA was prepared from different tissues with an RNAprep pure Plant Kit (TIANGEN, China), according to the manufacturers’ instructions. First strand cDNA was synthesized by using 1 µg total RNA and PrimeScript 1^st^ Strand cDNA Synthesis Kit (TaKaRa, Japan).

For alternative splicing analysis, One pair of specific primers was designed (Table S1) for each gene, to amplify the fragments of 55 *CsR2R3MYB* genes by RT-PCR with *TransStart™ FastPfu* DNA polymerase (TransGen, China). The amplified DNA fragments were purified using the TIANgel Midi Purification Kit (TIANGEN, China) and cloned with the Clone JET™ PCR Cloning Kit (Fermentas, China). Three independent clones for each of the different insert lengths were sequenced for sequence confirmation. Gene structures of the differently spliced transcripts were analyzed using GSDS (http://gsds.cbi.pku.edu.cn/) [84]. The ORFs were predicted for the transcripts that were cloned by using ORF Finder software (http://www.ncbi. nlm.nih.gov/gorf/gorf.html).

To analysis expression patterns of *CsR2R3MYB* genes, semi-quantitative RT-PCR was performed. *β-actin* gene (GenBank AB010922) was used as an internal control. The PCR primers were designed to avoid the conserved region and to amplify products of 150 to 300 bp long. Primer sequences were shown in detail in Table S2. Quantitative real-time PCR was carried out using the RealMasterMix (SYBR Green) kit (TIANGEN, China) and quantified the PCR amplification according to the manufacturers’ protocol. Amplification was performed on an iCycler iQ ™ multicolor real-time PCR detection system (Bio-Rad, hercules, USA) and the analysis of each type of sample was repeated four times. The analysis of relative mRNA expression data was performed using the 2^−ΔΔCt^ method [87]. Each expression profile was independently verified in 3 replicate experiments performed under identical conditions.

## Supporting Information

Figure S1 NJ phylogenetic tree of the 55 CsR2R3MYB members on the basis of complete protein sequences. The bootstrap values lower than 50 are not shown in the phylogenetic tree.
(TIF)

Figure S2 Phylogenetic relationships and subgroup designations in R2R3MYB proteins in cucumber, *Arabidopsis*, *Vitis*, *Oryza*, *Populus* and *Glycine*. Some functional clades and genes are listed to the right of the subgroups for reference.
(TIF)

Figure S3 Tissue-specific expression profiles of 55 cucumber *R2R3MYB* genes. *Cs* represented *CsR2R3MYB* assigned in Table 1.Total RNA was isolated from roots (R), stems (S), leaves (L), male flowers (MF), fruits (F) and tendrils (T). The cucumber *β-actin* gene (GenBank AB010922) was used to adjust cDNA concentrations. The PCR primers were designed to avoid the conserved region and to amplify products of 150 to 300 bp. Primer sequences were shown in detail in Table S2.
(TIF)

Figure S4 Expression patterns of cucumber abiotic-responsive *R2R3MYB* genes under different treatment conditions. A: NaCl (100mM); B: ABA (100 µM); C: Low temperature (4°C). *Cs* represented *CsR2R3MYB* assigned in Table 1.The cucumber *β-actin* gene (GenBank AB010922) was performed as an internal control. The PCR primers were designed to avoid the conserved region and to amplify products of 150 to 300 bp. Primer sequences were shown in detail in Table S2.
(TIF)

Table S1 Specific primers used for 55 *CsR2R3MYB* genes used in alternative splicing pattern analysis in this study.
(DOCX)

Table S2 Specific primers used for 55 cucumber *R2R3MYB* genes used in semi-quantitative RT-PCR in this study.
(DOCX)

Table S3 Expression patterns of 27 responsive *CsR2R3MYB* genes under three abiotic conditions.
(DOCX)
