# Supplementary figures and images for: Genome-Wide Identification and Characterization of R2R3MYB Family in Cucumis sativus
Source: PLoS One. 2012 Oct 23;7(10):e47576. doi: 10.1371/journal.pone.0047576 (PMC3479133; doi:10.1371/journal.pone.0047576)

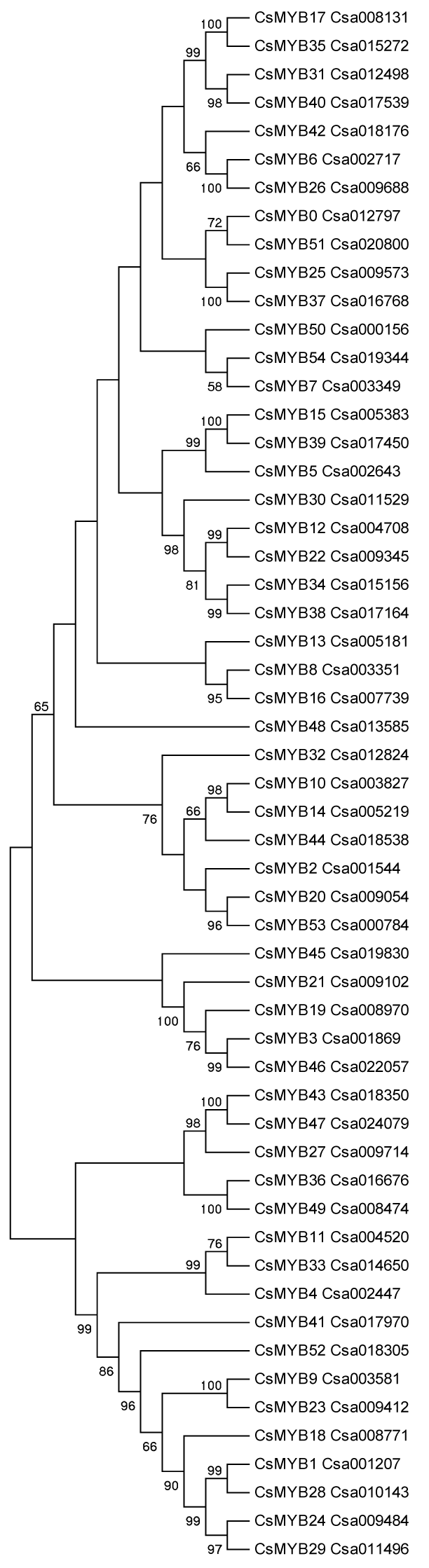

Supplement: Figure S1 — NJ phylogenetic tree of the 55 CsR2R3MYB members on the basis of complete protein sequences. The bootstrap values lower than 50 are not shown in the phylogenetic tree. (TIF) [file pone.0047576.s001.tif]

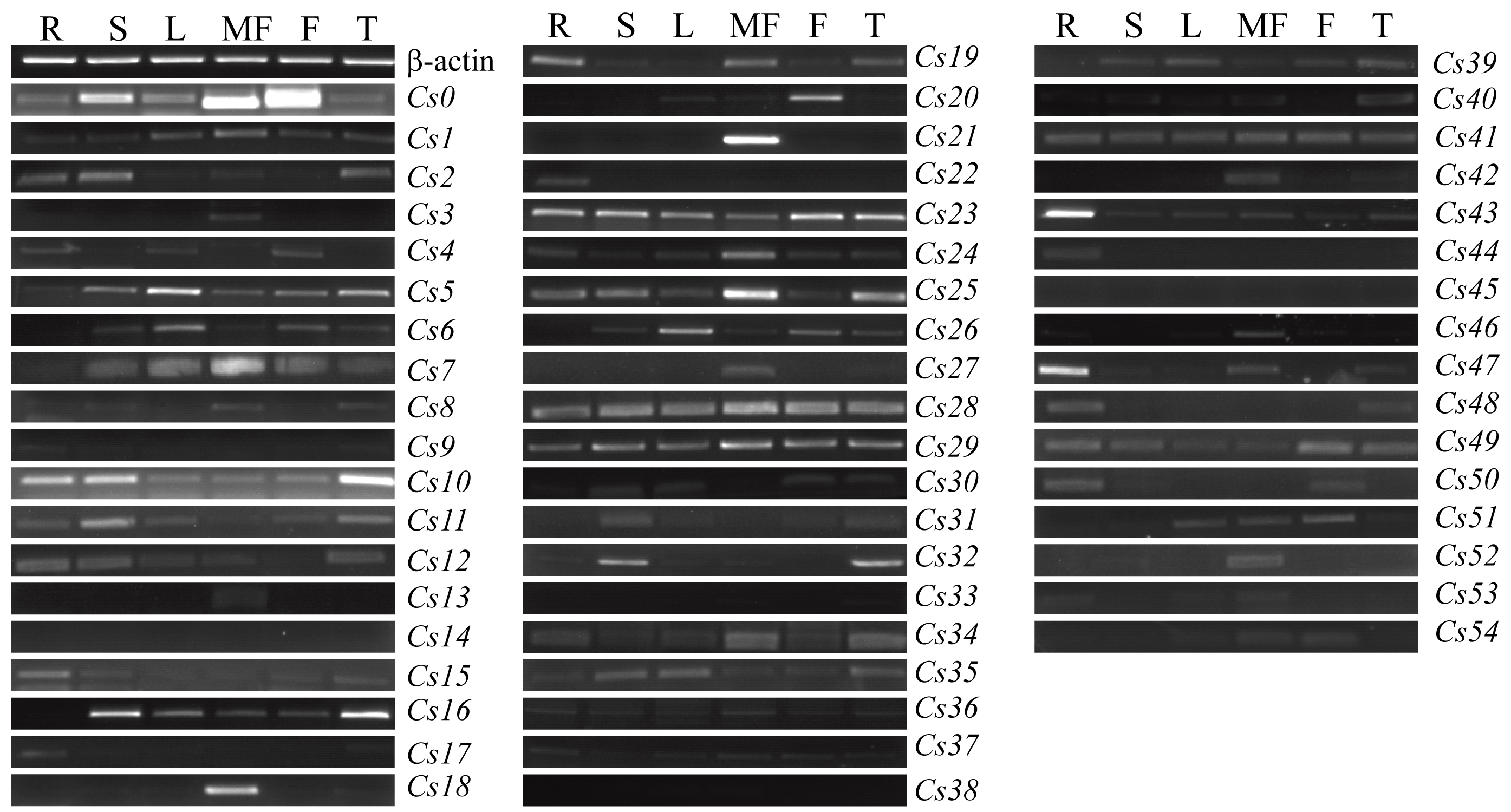

Supplement: Figure S3 — Tissue-specific expression profiles of 55 cucumber R2R3MYB genes. Cs represented CsR2R3MYB assigned in Table 1.Total RNA was isolated from roots (R), stems (S), leaves (L), male flowers (MF), fruits (F) and tendrils (T). The cucumber β-actin gene (GenBank AB010922) was used to adjust cDNA concentrations. The PCR primers were designed to avoid the conserved region and to amplify products of 150 to 300 bp. Primer sequences were shown in detail in Table S2. (TIF) [file pone.0047576.s003.tif]

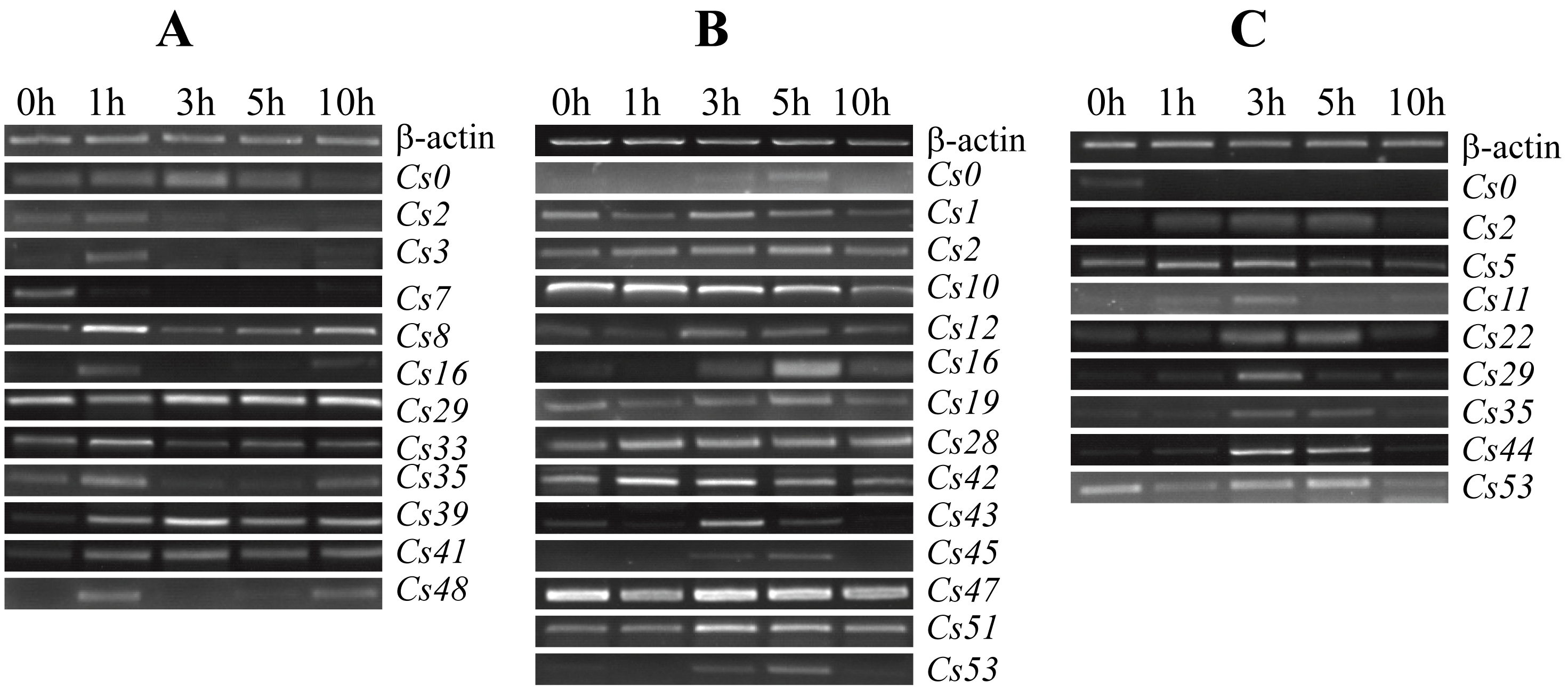

Supplement: Figure S4 — Expression patterns of cucumber abiotic-responsive R2R3MYB genes under different treatment conditions. A: NaCl (100mM); B: ABA (100 µM); C: Low temperature (4°C). Cs represented CsR2R3MYB assigned in Table 1.The cucumber β-actin gene (GenBank AB010922) was performed as an internal control. The PCR primers were designed to avoid the conserved region and to amplify products of 150 to 300 bp. Primer sequences were shown in detail in Table S2. (TIF) [file pone.0047576.s004.tif]
