# Supplementary material for: Genome-Wide Identification and Characterization of R2R3MYB Family in Cucumis sativus
Source: PLoS One. 2012 Oct 23;7(10):e47576. doi: 10.1371/journal.pone.0047576 (PMC3479133; doi:10.1371/journal.pone.0047576)
Supplement: Table S1 — Specific primers used for 55 CsR2R3MYB genes used in alternative splicing pattern analysis in this study. (DOCX) [file pone.0047576.s005.docx]

| *CsMYB* | Sense primer 5’-3’  Table S1. Specific primers used for 55 *CsR2R3MYB* genes used in alternative splicing pattern analysis in this study. | Antisense-primer 5’-3’ |
| --- | --- | --- |
| *0* | ATGGGAAGAACATCATCA | GCGTCGACTCAAATTAAGAAG |
| *1* | ATGTCCAAACAAACCGATCG | TCAGTCAATCCTCTGAACC |
| *2* | ATGGGAAGGCACTCTTGT | TTAAAGGGTATTTCCAAAAG |
| *3* | ATGGATGTTAAAATGAGAGG | TTAAACATCATCACCAAA |
| *4* | ATGCTTCTTAATGTTCCC | TTAGGAAGCTCCAACTCC |
| *5* | ATGGGAAGGCCTCCATGT | CTAGAACACCGGCGACAA |
| *6* | ATGGGAAGGTCTCCTTAC | TCAGAATCTCAGGAATTC |
| *7* | ATGGGGAGAGCTCCATGC | TTACACAAAATCAGCAAAGTCC |
| *8* | ATGGTTAGAGCTCCATTT | TCAAAAGAGATACAATTGGG |
| *9* | ATGATGAGAAGTTCGTCT | TTATTGAAACTCCGGATTTTCC |
| *10* | ATGGGGCGCCATTCTTGCTGT | TTATCCCATTATTATTGT |
| *11* | ATGTGCAGCAGAGGGCACT | TTAATTACCAGGTGGTGGTTTGGGG |
| *12* | ATGGGTAGAACTCCATGT | TCAAAAAAGAACATTGGTGGTGC |
| *13* | ATGGTAAGAATTCCTTCCTG | TTAGTAAAGTGGTTCCTC |
| *14* | ATGGGACGCAATTCTTGT | TCATATGTGATGAAAGATTCC |
| *15* | ATGGGAAGACCACCTTGT | CTAATTAAAGCTGAAAGG |
| *16* | ATGGTGCGAGCTCCTGTT | CTAGCAGAACAAATCACAG |
| *17* | ATGGGAAGGCAACCATGT | TTACAAGCCATAAACCCAAG |
| *18* | ATGCCGTCTGTACACCCC | TTATTTTTCATATTCAGCC |
| *19* | ATGGAAAGTGTTAGAAAAAG | TTAGAAATTCCACAAGCTCTCCG |
| *20* | ATGGGACACCATTCTTGC | CTATGCATCCCAGCTTGA |
| *21* | ATGGAAGAAGATTTGATTC | TTATTCAGCATTGAGAAGTTGC |
| *22* | ATGGGGAGAGCTCCTTGC | TCAAATTATCATCGAAGG |
| *23* | ATGAACATTCACAATTTT | TTAACCAAATCCCCGATTTTCCG |
| *24* | ATGGCTACCATCACTGAC | CTAATCGATCTTTCTTATTC |
| *25* | ATGGGAAGATCCCCTTGC | CTATGAAGTAGAACCCAT |
| *26* | ATGGGAAGGTCACCTTGT | TTAGAAAATTGAAGAATCAG |
| *27* | ATGGAAGGAAAAGAAGAGC | TCATTCTCTGAAACCTCCC |
| *28* | ATGGCGCTTACCCGTAAA | TTAAAAGCTAACGTTCTTAATCCC |
| *29* | ATGGATTCTGTTGGGAAG | CTAATCGATCTTGCTTATTCCC |
| *30* | ATGGGAAGAGCTCCATGT | TTAAAACAACATGCTGCA |
| *31* | ATGGGAAGGCAACCTTGT | TCAGTTTTTTTCTGCAATGCTC |
| *32* | ATGGGTCATCACTCTTGT | TTATAGAGATGTAGGGAA |
| *33* | ATGGCTGGGGTTGCAGGGGAT | TCACTGCTCAACCCCAAG |
| *34* | ATGGGAAGAGCTCCTTGT | TCACTTTGTACTTCTCTGTTC |
| *35* | ATGGGAAGACAGCCATGT | TCAAATTAATCCACAAGGCC |
| *36* | ATGGAAAGACAAAAAGGG | CTAGTTCAATGAAGGAGGAG |
| *37* | ATGGGTCGTTCTCCTTGT | TCACTTCATCTCCAAGCTTC |
| *38* | ATGGGAAGATCTCCTTGT | TCAGTACATGAAATTTTCTAG |
| *39* | ATGGGTAGGCCTCCTTGCT | TCATAGAAGATTATCCAAC |
| *40* | ATGGGCAGACAACCTTGCT | CTAGTGCTTCCCTTCCAT |
| *41* | ATGGAGGGCACCGCCGCT | CATAAATGCCCCATTTGC |
| *42* | ATGTCAGGATCGCCAACG | CCTCAGGCGTTGGCGATC |
| *43* | ATGGTTGAAGGACAGGAAATC | TCAATAGAAAAAATTCTGATTAGGC |
| *44* | ATGGTACGTGAAACGACG | TCAAATATTCATAAAACC |
| *45* | ATGACACCAAACACCCCG | TTAGCAAATTCCTGGCAAG |
| *46* | ATGGACGGAAATAAAGAC | TTAATTCAATTGCTGCTGC |
| *47* | ATGGGAATACAAGAAGAAAG | TTAGTTTTGAGTTATTAAAAC |
| *48* | ATGGCTAAGTGTTGTTGT | CTACAAGTATATGCCCTC |
| *49* | ATGGAGGAAAGAAGATCA | TCAGTTCTCCGGTGAGCT |
| *50* | ATGGTGAGAGCTCCTTGT | TCAAATGAGTGTGCCAAAAG |
| *51* | ATGGGGAGAACGCCGTGT | TCAACGACATTCTTCTCC |
| *52* | ATGGTTTTGATCGGAATCG | CTATGGAGTTTCTTGGGC |
| *53* | ATGAAGAAGGTGAAGAGAGG | AAGGATGGAATTTGGCGG |
| *54* | ATGGGGAGAGCTCCATGCT | TTCTTGCAACTCTCCATC |
