# Supplementary material for: Genome-Wide Identification and Characterization of R2R3MYB Family in Cucumis sativus
Source: PLoS One. 2012 Oct 23;7(10):e47576. doi: 10.1371/journal.pone.0047576 (PMC3479133; doi:10.1371/journal.pone.0047576)
Supplement: Table S2 — Specific primers used for 55 cucumber R2R3MYB genes used in semi-quantitative RT-PCR in this study. (DOCX) [file pone.0047576.s006.docx]

Table S2 Specific primers used for 55 cucumber *R2R3MYB* genes used in semi-quantitative RT-PCR in this study.

| *CsMYB* | Sense primer 5’-3’ | Antisense-primer 5’-3’ |
| --- | --- | --- |
| *0* | CTTGAGCAATGGTGTAGG | ACCATTCCATGATAACCC |
| *1* | GGAGAAGGAGGAGAGTAA | AATCCTCTGAACCACGAC |
| *2* | CCGGAAGACATGAAATGG | TATACAGCAGCATCGGAG |
| *3* | TCCCACAACTTCGTCATC | CCCATTCTCCAATACCTC |
| *4* | CCAACGCATCCTCTCTTT | GCTCCAACTCCAAGGAAA |
| *5* | AGAGTGTGAATTCGTGGG | GCGACAACTCCATCATCT |
| *6* | TTCCTTCTTCTTCAGGCG | ACCAAGAATTGTGTCCGG |
| *7* | CATATACGGCGGCAATGG | AAAGTCCAAACCTTCAGG |
| *8* | GCCCTCCAACACCATTAT | AGTGTGATTGAAGCCTCC |
| *9* | ATTGACCGCGTTGACTCT | CCATAAACGTCGTCGTCA |
| *10* | AATTCGGTGCCGAGTTTG | ACTGCTCCGTTGTTGTTG |
| *11* | CCGATAACGCAGTCAAGA | GGTGGTTTGGGGATGAAT |
| *12* | AGATCGTTGTCGTCGTCA | TTTCCTCGAGCCCATAGT |
| *13* | ATCTTTTGGCTCAGCTGC | TTTTCGTGTTCTCGAGGC |
| *14* | AGCATCTGGGTTGACTTC | CAGACGATTCCCATGAAG |
| *15* | TCAGAATCAAGCAGCAGC | ATACTGAGGTGTTGGTGC |
| *16* | GCTCATGTTTGTCCCAAC | GGTCGTAGGCTGTTTCAT |
| *17* | GTGCTTCTTCATCTTCAAC | ATTGGCATTACCTCCCTC |
| *18* | GAGCGATTGGGATGAGAT | GCCATGTAATTCCTCACC |
| *19* | CAGAGCTGTGGAGGTTAT | CACAAGCTCTCCGACTTA |
| *20* | AATTATGCGAGGTTGGCG | CTATGCATCCCAGCTTGA |
| *21* | ACACTCCACAAAGCAGCA | TGCATAGACCAGAGATCC |
| *22* | GCCGGCGTTATTGATGAT | AAGAACTCATTTCCCCCC |
| *23* | AATCACACTTCCGACGTC | CGTCGTCGTCATATACTC |
| *24* | ACACAACCTCCATCGATC | GTTTTACGACTGCGTTGG |
| *25* | GGGAGTCCATGTGAAAAC | AGAACCCATAGCACTTGC |
| *26* | GAGAATGTACGAACGGTG | GATGGAGAAGAATTGACC |
| *27* | GGGAAGAACTGACAACGA | CATTCTCTGAAACCTCCC |
| *28* | AGCATGACGACAACAGAG | GTTCTTAATCCCACCAGC |
| *29* | GGTATGGGTCGGATTCAA | CAACAGCGTTTCTGATGG |
| *30* | GCCAAAACCCAGAGTCAT | CATATCATGTCTGCTGGG |
| *31* | CCAAGGAAGCTCAACTGA | TAAAGTCCCCCAAAGAAC |
| *32* | TGCTTAGCCCTCAAATGG | TATTAGCTCCTCCTCCTC |
| *33* | TTGATCTCTCACCCACAC | ACTGCTCAACCCCAAGAA |
| *34* | CCCCATCAAAATCACTCC | GCTCCATCATCTCTTCCT |
| *35* | TCGAGCCTAAAACACCAC | TCCTCATGATCACCAACC |
| *36* | AGCACCAAACCACATTCC | TGAAGGAGGAGATCGATC |
| *37* | TCTCCTTCACTGCTACTG | ATTCCCAGTTGAAGTACC |
| *38* | CCCTGTCTTGTCCAAACA | CTCAAGATTAGCAGCTGG |
| *39* | GGCAGTGGAGGAAAAGTA | TCTCCATGAAGCCATTCC |
| *40* | CAGCAGCAACAACAACCA | GCTTCCCTTCCATTTCTG |
| *41* | CCTCATCAGTGTGGTCAT | CCCATTTGCATCTGAGAACC |
| *42* | TGACTATACAGCAGCCAC | TCCATGACGGAGGAGTTT |
| *43* | GAAGTGGGTGGTGATCAA | GATTAGGCTGAGCTAAGC |
| *44* | CTACTTCAGACTCTGCAG | TTGAAGTAGCTCCGAACC |
| *45* | GCAGCAATGCCATCAAAC | ATTCCTGGCAAGTTGTCC |
| *46* | TCATCCATGTCGGAGTTG | GCTGCTGCAAAAACCATA |
| *47* | GGGAATCCGTCAGAAGAA | AACAACACCCTCTCCATG |
| *48* | ATGGATCCAACGGCTGTA | AGATATTGATCCAACCCC |
| *49* | GCAATTTCCACACTCAGTC | GCGGCTCTAGTTCAAATA |
| *50* | CTCAAAAAAGGGCCATGG | TTATCGCTGCAATGGCTG |
| *51* | GACTGATGATGGCGAGAA | ACGACATTCTTCTCCTCC |
| *52* | GGTGGAGAGCGAAAAGAA | ATGGAGTTTCTTGGGCTG |
| *53* | GTGGGTATGGTTGCAGAA | GGCGGAATATTTGACATGGG |
| *54* | AATTGGATGGAGAGGAAG | GCTCATTTCTTCTTCCCC |
