# Supplementary material for: Genome-Wide Identification and Characterization of R2R3MYB Family in Cucumis sativus
Source: PLoS One. 2012 Oct 23;7(10):e47576. doi: 10.1371/journal.pone.0047576 (PMC3479133; doi:10.1371/journal.pone.0047576)
Supplement: Table S3 — Expression patterns of 27 responsive CsR2R3MYB genes under three abiotic conditions. (DOCX) [file pone.0047576.s007.docx]

Table S3 Expression patterns of 27 responsive *CsR2R3MYB* genes under three abiotic conditions.

| *CsMYB* | NaCl stress | ABA treatment | Low temperature stress |
| --- | --- | --- | --- |
| *0* | I | I | R |
| *1* |  | R |  |
| *2* | I | I | I |
| *3* | I |  |  |
| *5* |  |  | I |
| *7* | R |  |  |
| *8* | I |  |  |
| *10* |  | R |  |
| *11* |  |  | I |
| *12* |  | I |  |
| *16* | I | R |  |
| *19* |  | R |  |
| *22* |  |  | I |
| *28* |  | I |  |
| *29* | R |  | I |
| *33* | I |  |  |
| *35* | I |  | I |
| *39* | I |  |  |
| *41* | I |  |  |
| *42* |  | I |  |
| *43* |  | I |  |
| *44* |  |  | I |
| *45* |  | I |  |
| *47* |  | I |  |
| *48* | I |  |  |
| *51* |  | I |  |
| *53* |  | I | R |

I, induced; R, repressed.
